# An Observation-Driven Random Parameter INAR(1) Model Based on the Poisson Thinning Operator

**DOI:** 10.3390/e25060859

**Published:** 2023-05-27

**Authors:** Kaizhi Yu, Tielai Tao

**Affiliations:** School of Statistics, Southwestern University of Finance and Economics, Chengdu 611130, China; yukz@swufe.edu.cn

**Keywords:** integer-valued time series, thinning operator, observation-driven, ergodicity, interval estimation

## Abstract

This paper presents a first-order integer-valued autoregressive time series model featuring observation-driven parameters that may adhere to a particular random distribution. We derive the ergodicity of the model as well as the theoretical properties of point estimation, interval estimation, and parameter testing. The properties are verified through numerical simulations. Lastly, we demonstrate the application of this model using real-world datasets.

## 1. Introduction

Integer-valued time series data are prevalent in both scientific research and various socioeconomic contexts. Examples of such data encompass the annual number of companies listed on stock exchanges, the monthly usage of hospital beds in specific departments, and the yearly frequency of major earthquakes or tsunamis. However, traditional continuous-valued time series models are unable to precisely capture the unique characteristics of integer-valued data, resulting in only approximations through continuous-valued models. This shortcoming may lead to model mis-specification, posing challenges in statistical inference. Consequently, the modeling and analysis of integer-valued time series data have increasingly gained attention within academia. Amongst the extensive range of integer-valued time series models, thinning operator models have attracted considerable interest from scholars due to their resemblance to Autoregressive Moving Average (ARMA) models in continuous-valued time series theory. Thinning operator models replace multiplication in ARMA models with the binomial thinning operator, which was initially introduced by Steutel and Van Harn [1]:

(1) $$\phi \circ Y_{i}=\sum_{i=1}^{Y_{i}}B_{i},\;$$

where $\left\{Y_{i}\right\}$ refers to a count series and $\left\{B_{i}\right\}$ represents a Bernoulli random variable sequence that independent of $\left\{Y_{i}\right\}$, satisfying the condition $P\left(B_{i}=1\right)=1-P\left(B_{i}=0\right)=\phi$. Building upon this concept, Al-Osh and Alzaid [2] developed the first-order Integer-valued Autoregressive (INAR(1)) model, for $t\in {\mathbb{N}}^{+}$:

(2) $$Y_{t}=\phi \circ Y_{t-1}+Z_{t},\;$$

where $Z_{t}$ is considered the innovation term entering the model during period t. Its marginal distribution aligns with a Poisson distribution, exhibiting an expected value of λ, thereby giving rise to the nomenclature of the Poisson INAR(1) model. An intuitive interpretation of this model is that, within a hospital setting, the number of in-patients in period t comprises patients from period t−1 who have not yet been discharged, along with patients newly admitted in period t. Given that $B_{i}$ adheres to a Bernoulli distribution, the binomial thinning operator can exclusively express the {0,1} to {0,1} excitation states. However, the binomial thinning operator does not represent the sole available option for thinning operators. Latour [3] expanded the distribution of $B_{i}$ in Equation (1) to encompass any non-negative integer-valued random variable, thus establishing the Generalized Integer-valued Autoregressive (GINAR) model and providing conditions for model stationarity. Furthermore, the ϕ in Equation (1) need not be a fixed constant. Joe [4] and Zheng, Basawa, and Datta [5] constructed the Random Coefficient Thinning Operator (RCINAR(1)) model by permitting the parameter ϕ in the INAR(1) model to follow a specified random distribution. Gomes and Castro [6] generalized the thinning operator in RCINAR(1) to GINAR(1) model, culminating in the development of the Random Coefficient Generalized Integer-valued Autoregressive model. Weiß and Jentsch [7] proposed a bootstrap estimation method based on the INAR model to facilitate the introduction of semi-parametric structures within the INAR model, in turn reducing model assumptions and augmenting model generalization capabilities. Kang, Wang, and Yang [8] mixed the binomial thinning operator with the operator introduced by Pegram [9], resulting in the development of a novel INAR model capable of addressing equi-dispersed, under-dispersed, over-dispersed, zero-inflated, and multimodal integer-valued time series data. Salinas, Flunkert, Gasthaus, and Januschowski [10] proposed a new method for time series forecasting based on autoregressive recurrent neural network models. Huang, Zhu, and Deng [11] mixed quasi-binomial distribution operators with generalized Poisson operators, thus equipping the INAR model with the ability to describe structural changes in the data generation processes. Mohammadi, Sajjadnia, Bakouch, and Sharafi [12] incorporated innovation terms conforming to the Poisson-Lindley distribution, thereby enhancing the INAR(1) model’s capacity to capture {0,1} inflated integer-valued time series data. For further discussion on thinning operator models, Scotto, Weiß, and Gouveia [13] provide a comprehensive review article.

The thinning operator models previously mentioned presuppose that ϕ is independent of other variables, thereby neglecting the dynamic features of the coefficient ϕ in INAR models. To tackle this limitation, Zheng and Basawa [14] proposed a first-order observation-driven integer-valued autoregressive process. Triebsch [15] introduced the first-order Functional Coefficient Integer-valued Time Series model based on the thinning operator, in which the coefficient $\phi_{t}$ during period t is a measurable function of the previous observation $Y_{t-1}$. Furthermore, Montriro, Scotto, and Pereira [16] presented the Self-Exciting Threshold Integer-valued Time Series model (SETINAR) in which the coefficient $\phi_{t}$ during period t assumes diverse values contingent on the varying observations in prior limited periods. Building on the geometric thinning operator (alternatively known as the negative binomial thinning operator) proposed by Ristić, Bakouch, and Nastić [17], Yu, Wang, and Yang [18] introduced an INAR(1) model encompassing observation-driven parameters.

With respect to integer-valued time series models featuring observation-driven parameters, existing studies primarily focus on binomial and geometric thinning operators. However, the binomial thinning operator cannot represent one-to-many excitation states, and both binomial and geometric thinning operators exhibit limited descriptive capacity for locally non-stationary phenomena and extreme values in real data. Consequently, this paper employs a Poisson thinning operator, defined as follows:

(3) $$\phi_{t}⊖Y_{t}=\sum_{i=1}^{Y_{t}}B_{i}^{\left(t\right)},\;$$

where, $\left\{B_{i}^{\left(t\right)}\right\}$ is independent of $Y_{t}$ and constitutes an independent and identically distributed Poisson random variable sequence with an intensity parameter $\phi_{t}>0$. The probability mass function is expressed by:

$$\mathbb{P}\left(B_{i}^{\left(t\right)}=x\right)=\frac{\phi_{t}^{x}}{x!}\exp\left(-\phi_{t}\right),$$

where $\left\{B_{i}^{\left(t\right)}\right\}$ and $Y_{t}$ are mutually independent. Leveraging this thinning operator, the INAR(1) model in this study is formulated as follows:

$$Y_{t}=\phi_{t}⊖Y_{t-1}+Z_{t},$$

where the sequence $\left\{Z_{t}\right\}$ comprises independent and identically distributed non-negative integer-valued random variables, which are independent of $\left\{B_{i}^{\left(t\right)}\right\}$ and ${\left\{Y_{s}\right\}}_{s<t}$. Furthermore, diverging from the parameters set forth by Yu, Wang, and Yang [18], we posit that $\phi_{t}$ correlates with the previous observation $Y_{t-1}$, and given $Y_{t-1}$, $\phi_{t}|Y_{t-1}$ may still conform to a specific non-negative probability distribution. In Section 2, we will demonstrate that if the expectation of this non-negative discrete probability distribution falls below 1, it does not affect the model’s ergodicity. Simultaneously, due to instances where $\phi_{t}|Y_{t-1}$ occasionally exceeds 1, the autoregressive model exhibits non-stationary features or generates extreme values within specific periods—all without compromising its overall stationarity. In comparison to existing research, this setting offers the advantage of simultaneously illustrating one-to-many excitation states and observation-driven and time-varying parameter structures, as well as localized non-stationary features or extreme values. For example, in public health, a patient with an infectious disease may not transmit the illness to others or could potentially infect one or multiple individuals, indicating one-to-many excitation states. As the number of infections fluctuates, local epidemic prevention policies may undergo changes, consequently modifying the disease’s transmissibility and reflecting the time-varying and observation-driven characteristics of the coefficient. During particular periods of rapid infectious disease spread, the majority of infected individuals are likely to infect more than one other person, resulting in infection data that exhibit extreme values or localized non-stationary characteristics.

The organization of this paper is as follows: in Section 2, we introduce the integer-valued time series model featuring observation-driven coefficients under investigation and outline its essential statistical properties. In Section 3, we describe the estimation and testing methods pertinent to this model and present asymptotic results. Section 4 provides numerical simulation outcomes of these techniques, elaborating on the performance of the estimation and testing approaches across diverse settings and sample conditions. Section 5 demonstrates the application of the proposed model using real-world data. Finally, Section 6 offers a summary and discussion.

## 2. Model Construction and Basic Properties

For the time series $\left\{Y_{t}\right\}$, consider the following data generating process:

(4) $$Y_{t}=\phi_{t}⊖Y_{t-1}+Z_{t}$$

Given $Y_{t-1}$, $\phi_{t}$ may be fixed as:

$${\frac{\exp\left[\nu \left(Y_{t-1};\beta \right)\right]}{1+\exp\left[\nu \left(Y_{t-1};\beta \right)\right]}}_{.}$$

Alternatively, $\left\{\phi_{t}\right\}$ could represent an independent random variable sequence with a conditional expectation of:

(5) $$E\left(\phi_{t}|Y_{t-1}\right)={\frac{\exp\left[\nu \left(Y_{t-1};\beta \right)\right]}{1+\exp\left[\nu \left(Y_{t-1};\beta \right)\right]}}_{,}$$

where β is an ℓ-dimensional parameter vector, the function ν(·;·) belongs to a specific parametric family of functions $G\left\{\nu \left(Y_{t-1};\beta \right);\beta \in \Theta \right\}$, and Θ is a compact subset of ${\mathbb{R}}^{\ell }$. β is an interior point of Θ and ν(y;β) is thrice continuously differentiable with respect to β. The conditional variance is given by $Var\left(\phi |Y_{t-1}\right)=\sigma_{\phi_{t}|Y_{t-1}}^{2}$. Additionally, $\left\{Z_{t}\right\}$ comprises an independent and identically distributed non-negative integer-valued random variable sequence with a probability mass function $f_{z}$ with expectation $E\left(Z_{t}\right)=\lambda <\infty$ and variance $Var\left(Z_{t}\right)=\sigma_{Z}^{2}<\infty$. Furthermore, $\left\{Z_{t}\right\}$ is independent of $\left\{Y_{t}\right\}$.

**Remark** **1.** *Integer-valued probability distributions that align with the settings of* $Z_{t}$ *are common, with typical examples being Poisson and geometric distributions. This paper employs a Poisson distribution in the numerical simulation section.*

**Remark** **2.** *There are numerous functions that align with the setting of* ν(·;·)*, with the most typical being the linear function* $\nu \left(Y_{t-1};\beta \right)=\beta_{0}+\beta_{1}Y_{t-1}$*. In this paper’s numerical simulation section, a linear function setting will be adopted.*

**Remark** **3.** *From model (4), it is evident that* $\left\{Y_{t}\right\}$ *is a Markov chain defined on the set of natural numbers* ℕ*, with a one-step-ahead transition probability:*

(6) $$\mathbb{P}\left(Y_{t}=y_{t}|Y_{t-1}=y_{t-1}\right)=\int^{\;}\mathbb{P}\left(Y_{t}=y_{t}|Y_{t-1}=y_{t-1},\phi_{t}=\phi \right)\mathbb{P}\left(\phi_{t}=\phi |Y_{t-1}=y_{t-1}\right)d\phi \;=\int^{\;}\overset{y_{t}}{\underset{k=0}{\sum }}\frac{{\left(\phi y_{t-1}\right)}^{k}}{k!}\exp\left(-\phi y_{t-1}\right)f_{z}\left(y_{t}-k\right)\mathbb{P}\left(\phi_{t}=\phi |Y_{t-1}=y_{t-1}\right)d\phi$$

Based on the above model construction, we can obtain the conditional moments for Model (4). Starting from these conditional moments, we can construct estimating equations to estimate the unknown parameters in the model:

**Property** **1.** *for* t≥1

*(i)*  $E\left(Y_{t}|Y_{t-1}\right)=\frac{\exp\left[\nu \left(Y_{t-1};\beta \right)\right]}{1+\exp\left[\nu \left(Y_{t-1};\beta \right)\right]}Y_{t-1}+\lambda$,
*(ii)*  $Var\left(Y_{t}|Y_{t-1}\right)=\frac{\exp\left[\nu \left(Y_{t-1};\beta \right)\right]}{1+\exp\left[\nu \left(Y_{t-1};\beta \right)\right]}Y_{t-1}+\sigma_{Z}^{2}+\sigma_{\phi_{t}|Y_{t-1}}^{2}$,
*(iii)*  $Cov\left(Y_{t},Y_{t-1}\right)=E\left(\frac{\exp\left[\nu \left(Y_{t-1};\beta \right)\right]}{1+\exp\left[\nu \left(Y_{t-1};\beta \right)\right]}Y_{t-1}^{2}\right)-E\left(\frac{\exp\left[\nu \left(Y_{t-1};\beta \right)\right]}{1+\exp\left[\nu \left(Y_{t-1};\beta \right)\right]}Y_{t-1}\right)E\left(Y_{t-1}\right)$.

Ergodicity is crucial for the convergence of parameter estimation, as presented in the following property:

**Property** **2.** *If* $\underset{y\in \mathbb{N}}{\sup}\nu \left(y;\beta \right)<\infty$*,* β∈Θ*, then the data generating process* $\left\{Y_{t}\right\}$ *defined by (4) is an ergodic Markov chain.*

**Remark** **4.** *In Property 2, since the form of the function* ν *is not determined, we cannot directly provide the conditions for the ergodicity of* $\left\{Y_{t}\right\}$*. However, for specific cases, such as* $\nu \left(Y_{t-1};\beta \right)=\beta_{0}+\beta_{1}Y_{t-1}$*, we can intuitively see that the stationary and ergodic property of the data generating process requires* $\beta_{1}\leq 0$ *at the very least, making the expected value of* $\phi_{t}$ *lower when* $Y_{t}$ *is higher and vice versa. From the proof of Property A1 in Appendix A, it can be observed that the ergodicity of* $\left\{Y_{t}\right\}$ *requires the existence of a constant* 0<m<1 *such that* $\frac{\exp\left(\beta_{0}+\beta_{1}Y_{t-1}\right)}{1+\exp\left(\beta_{0}+\beta_{1}Y_{t-1}\right)}<m$*; however, if* $\beta_{1}>0$*, then* $\frac{\exp\left(\beta_{0}+\beta_{1}Y_{t-1}\right)}{1+\exp\left(\beta_{0}+\beta_{1}Y_{t-1}\right)}$ *will increase with the rise of* $Y_{t}$*, making it impossible to determine a constant* m *that meets requirements*.

## 3. Parameter Estimation and Hypothesis Testing

In this section, we assume that the time series ${\left\{Y_{t}\right\}}_{t=1}^{T}$ satisfies the data-generating process defined by Equation (4), with $\theta_{0}=\left(\beta_{0}^{\prime },\lambda_{0}\right)$ as the true parameter vector of this process and $\theta =\left(\beta^{\prime },\lambda \right)$ as the unknown parameter vector to be estimated. In this paper, our primary focus is on two estimation methods: Conditional Least Squares (CLS) and Conditional Maximum Likelihood (CML). Additionally, we attempt to establish observation-driven interval estimation through estimating equations in CLS and observation-driven hypothesis testing through the framework of Empirical Likelihood (EL). Here, we first make assumptions about the data-generating process $\left\{Y_{t}\right\}$ and the function ν(y;β), assuming the existence of a neighborhood B of $\beta_{0}$ and a positive integrable function N(y), such that:

(A1) $\left\{Y_{t}\right\}$ is a strictly stationary and ergodic sequence.
(A2) 1≤i,j≤ℓ, $\left|\frac{𝜕\nu \left(y;\beta \right)}{𝜕\beta_{i}}\right|$ and $\left|\frac{{𝜕}^{2}\nu \left(y;\beta \right)}{𝜕\beta_{i}𝜕\beta_{j}}\right|$ are continuous with respect to β and dominated by N(y) on B, where N(y) is a positive integrable function.
(A3) $1\leq i,j,k\leq \ell ,\;\left|\frac{{𝜕}^{3}\nu \left(y;\beta \right)}{𝜕\beta_{i}𝜕\beta_{j}𝜕\beta_{k}}\right|$ are continuous with respect to β and dominated by N(y) on B, where N(y) is a positive integrable function.
(A4) ∃δ>0, such that $E{\left|Y_{t}\right|}^{8+\delta }<\infty$, $E{\left|N\left(Y_{t}\right)\right|}^{8+\delta }<\infty$.
(A5) $E\left(\frac{𝜕\nu \left(y;\beta \right)}{𝜕\beta }\cdot \frac{𝜕\nu \left(y;\beta \right)}{𝜕\beta^{\prime }}\right)$ is a full-rank matrix, i.e., of rank ℓ.
(A6) The parameters of ν(y;β) are identifiable, that is, if $\beta \neq \beta_{0}$, then $P_{\nu \left(Y_{t};\beta \right)}\neq P_{\nu \left(Y_{t};\beta_{0}\right)}$, where $P_{\nu \left(Y_{t};\beta \right)}$ represents the marginal probability measure of $\nu \left(Y_{t};\beta \right)$.

### 3.1. Conditional Least Squares Estimation

Let $S\left(\theta \right)=\overset{T}{\underset{t=2}{\sum }}{\left(Y_{t}-E\left(Y_{t}|Y_{t-1}\right)\right)}^{2}=\overset{T}{\underset{t=2}{\sum }}{\left(Y_{t}-\frac{\exp\left[\nu \left(Y_{t-1};\beta \right)\right]}{1+\exp\left[\nu \left(Y_{t-1};\beta \right)\right]}Y_{t-1}-\lambda \right)}^{2}$, where $\theta =\left(\beta^{\prime },\;\lambda \right)$. The CLS estimator is then given by:

$${\hat{\theta }}_{CLS}=argmin_{\theta }\left(S\left(\theta \right)\right).$$

Let $S_{t}\left(\theta \right)={\left(Y_{t}-E\left(Y_{t}|Y_{t-1}\right)\right)}^{2}$. The first-order condition equation is represented as follows:

(7) $$-\frac{1}{2}\frac{𝜕S_{t}\left(\theta \right)}{𝜕\theta }=0=M_{t}\left(\theta \right)={\left(m_{t1}\left(\theta \right),m_{t2}\left(\theta \right),\ldots ,m_{t\left(\ell +1\right)}\left(\theta \right)\right)}^{\prime },\;$$

where

$$m_{ti}\left(\theta \right)=\left(Y_{t}-\frac{\exp\left[\nu \left(Y_{t-1};\beta \right)\right]}{1+\exp\left[\nu \left(Y_{t-1};\beta \right)\right]}Y_{t-1}-\lambda \right)\frac{\exp\left[\nu \left(Y_{t-1};\beta \right)\right]}{{\left(1+\exp\left[\nu \left(Y_{t-1};\beta \right)\right]\right)}^{2}}\frac{𝜕\nu \left(y;\beta \right)}{𝜕\beta_{i}}Y_{t-1},\;1\leq i\leq \ell ,$$

$$m_{t\left(\ell +1\right)}\left(\theta \right)=Y_{t}-\frac{\exp\left[\nu \left(Y_{t-1};\beta \right)\right]}{1+\exp\left[\nu \left(Y_{t-1};\beta \right)\right]}Y_{t-1}-\lambda .$$

Thus, the estimating equation is given by $\overset{T}{\underset{t=1}{\sum }}M_{t}\left(\theta \right)=0$. Solving this equation provides the CLS estimate ${\hat{\theta }}_{CLS}$ for the parameter vector θ=(β,λ).

**Theorem** **1.** *Under assumptions (A1) to (A5), the CLS estimator* ${\hat{\theta }}_{CLS}$ *is a consistent estimator for the true parameter* $\theta_{0}$*, and it has an asymptotic distribution:*

$$\sqrt{T}\left({\hat{\theta }}_{CLS}-\theta_{0}\right)\overset{d}{\to }N\left(0,V^{-1}\left(\theta_{0}\right)W\left(\theta_{0}\right)V^{-1}\left(\theta_{0}\right)\right),$$

*where*

$$W\left(\theta_{0}\right)=E\left(M_{t}\left(\theta_{0}\right)M_{t}^{\prime }\left(\theta_{0}\right)\right),$$

$$V\left(\theta_{0}\right)=E\left(\frac{𝜕E\left(Y_{t}|Y_{t-1}\right)}{𝜕\theta }\cdot \frac{𝜕E\left(Y_{t}|Y_{t-1}\right)}{𝜕\theta^{\prime }}\right)-E\left(u_{t}\left(\theta_{0}\right)\frac{{𝜕}^{2}E\left(Y_{t}|Y_{t-1}\right)}{𝜕\theta 𝜕\theta^{\prime }}\right),$$

$$u_{t}\left(\theta_{0}\right)=Y_{t}-E\left(Y_{t}|Y_{\left(t-1\right)}\right).$$

### 3.2. Interval Estimation

Based on the estimating equations from the CLS estimation, we can construct observation-driven interval estimation and hypothesis testing. Let:

$$H\left(\theta \right)={\left(\overset{T}{\underset{t=2}{\sum }}M_{t}\left(\theta \right)\right)}^{\prime }{\left(\overset{T}{\underset{t=2}{\sum }}M_{t}\left(\theta \right)M_{t}{\left(\theta \right)}^{\prime }\right)}^{-1}{\left(\overset{T}{\underset{t=2}{\sum }}M_{t}\left(\theta \right)\right)}_{.}$$

We can then obtain the following theorem:

**Theorem** **2.** *Under assumptions (A1)–(A5), as* T→∞*,*

(8) $$H\left(\theta_{0}\right)\overset{d}{\to }\chi^{2}\left(\ell +1\right).$$

**Remark** **5.** *From Equation (8), we can construct an interval estimation for* $\theta_{0}$*:*

$$\left\{\theta |H\left(\theta \right)\leq C_{\alpha }\right\},$$

*where* $C_{\alpha }$ *satisfies that for* 0<α<1*,* $\mathbb{P}\left(\chi_{\ell +1}^{2}\leq C_{\alpha }\right)=\alpha$*. From the perspective of hypothesis testing, this serves as an acceptance region for testing the null hypothesis* ${ℍ}_{0}:\theta_{0}=\theta$*. If* $H\left(\theta \right)>C_{\alpha }$*; then the null hypothesis is rejected.*

### 3.3. Empirical Likelihood Test

In the following, we introduce hypothesis testing based on empirical likelihood estimation. First, we provide a brief introduction to the empirical likelihood (EL) method. Initially proposed by Owen [19] for providing interval estimations for expectation, the EL method was later extended to estimating equation estimation by Qin and Lawless [20]. For T observations $y_{1},y_{2},\ldots ,y_{T}$ of a random variable Y with distribution F, the empirical likelihood ratio is defined as:

$$R\left(F\right)=\frac{L\left(F\right)}{L\left(F_{T}\right)}=\overset{T}{\underset{t=1}{\prod }}Tp_{t},$$

where $L\left(F\right)=\overset{T}{\underset{t=1}{\prod }}p_{t}$ is the nonparametric likelihood function, $p_{t}=dF\left(y_{t}\right)=\mathbb{P}\left(Y=y_{t}\right)$, and $F_{T}\left(y\right)=\frac{1}{T}\overset{T}{\underset{t=1}{\sum }}1_{\left\{y_{t}\leq y\right\}}$ is the empirical distribution function of the random variable Y, $dF_{T}=\frac{1}{T}$, ∀t∈T. Under constraints $\overset{T}{\underset{t=1}{\sum }}p_{t}=1$ and $p_{t}\geq 0,\forall t$, $F_{T}$ maximizes L(F), so R(F)≤1.

Suppose we are interested in the parameter vector θ, which satisfies the estimating equation $E\left(M_{t}\left(\theta \right)\right)=0$. We need to add a new constraint for $p_{t}$: $\overset{T}{\underset{t=1}{\sum }}p_{t}M_{t}\left(\theta \right)=0$. Based on this, we can establish the profile empirical likelihood ratio function:

$$ℛ\left(\theta \right)=\sup{\left\{\overset{T}{\underset{t=1}{\prod }}Tp_{t}:p_{t}\geq 0,\overset{T}{\underset{t=1}{\sum }}p_{t}=1,\overset{T}{\underset{t=1}{\sum }}p_{t}M_{t}\left(\theta \right)=0\right\}}_{.}\;$$

The profile empirical likelihood ratio function can be solved using the Lagrange multiplier method. Let:

$$ℒ\left(\theta \right)=\overset{T}{\underset{t=1}{\sum }}\log\left(p_{t}\right)+𝓀\left(\overset{T}{\underset{t=1}{\sum }}p_{t}-1\right)+\gamma^{\prime }T\overset{T}{\underset{t=1}{\sum }}p_{t}M_{t}\left(\theta \right),$$

where 𝓀 and γ are Lagrange multipliers. It can be proved that when ℒ(θ) is maximized, 𝓀=T, and:

$$p_{t}=\frac{1}{T}\cdot {\frac{1}{\gamma^{\prime M_{t}}\left(\theta \right)}}_{.}$$

Here, as a function of θ, γ=γ(θ) is the solution to the following equation:

(9) $$\overset{T}{\underset{t=1}{\sum }}\frac{M_{t}\left(\theta \right)}{1+\gamma^{\prime }M_{t}\left(\theta \right)}=0,$$

substituting this into $p_{t}$ and R(F), we find:

$$R\left(F\right)=\overset{T}{\underset{t=1}{\prod }}{\frac{1}{1+\gamma {\left(\theta \right)}^{\prime }M_{t}\left(\theta \right)}}_{.}$$

Thus, the log empirical likelihood ratio function can be defined as:

$${ℒ}_{E}\left(\theta \right)=-\log\left(ℛ\left(\theta \right)\right)=\overset{T}{\underset{t=1}{\sum }}log\left[1+\gamma {\left(\theta \right)}^{\prime }M_{t}\left(\theta \right)\right].$$

The empirical likelihood estimate is then given by:

$${\hat{\theta }}_{EL}=argmin_{\theta }\left({ℒ}_{E}\left(\theta \right)\right).$$

The corresponding γ is denoted by $\hat{\gamma }\left({\hat{\theta }}_{EL}\right)$.

**Remark** **6.** *Given that* $0\leq p_{t}\leq 1$ *for all* t∈T*, it can be deduced that* ${ℒ}_{E}\left(\theta \right)=-\log\left(\overset{T}{\underset{t=1}{\prod }}p_{t}\right)\geq 0$.

**Remark** **7.** *Since the number of estimating equations matches the number of parameters to be estimated (also known as just-identified in some econometrics literature), and* ${\hat{\theta }}_{CLS}$ *is the solution to the estimating equation* $\overset{T}{\underset{t=1}{\sum }}M_{t}\left(\theta \right)=0$*, it follows from Chen and Keilegom [21] that:*

$${\hat{\theta }}_{EL}={\hat{\theta }}_{CLS}.$$

Therefore, we will omit empirical likelihood estimation in the point estimation segment in the numerical simulation section.

**Theorem** **3.** *Under assumptions (A1)–(A5), let* $\theta ={\left(\theta_{1}^{\prime },\theta_{2}^{\prime }\right)}^{\prime }$*, where* $\theta_{1}$ *and* $\theta_{2}$ *are* q×1 *and* (ℓ+1−q)×1*-dimensional parameter vectors to be estimated, respectively. For the hypothesis* ${ℍ}_{0}:\theta^{\left(1\right)}=\theta_{0}^{\left(1\right)}$*, a test statistic can be constructed as follows:*

$${ℒ}_{E}\left(\theta_{0}^{\left(1\right)},{\tilde{\theta }}_{EL}^{\left(2\right)}\right)-{ℒ}_{E}\left({\hat{\theta }}_{EL}^{\left(1\right)},{\hat{\theta }}_{EL}^{\left(2\right)}\right)\overset{d}{\to }\chi^{2}\left(q\right),$$

*where* $\left({\hat{\theta }}_{EL}^{\left(1\right)},{\hat{\theta }}_{EL}^{\left(2\right)}\right)={\hat{\theta }}_{EL}$*, and* ${\tilde{\theta }}_{EL}^{\left(2\right)}$ *is the estimate obtained by minimizing* ${ℒ}_{E}\left(\theta_{0}^{\left(1\right)},\theta^{\left(2\right)}\right)$ *concerning* $\theta^{\left(2\right)}$.

**Remark** **8.** *As Remark 7 indicates, in a just-identified situation,* ${\hat{\theta }}_{EL}={\hat{\theta }}_{CLS}$ *and* ${ℒ}_{E}\left({\hat{\theta }}_{CLS}\right)=0$*. Thus, the conclusion of Theorem 3 can be further simplified as:*

$${ℒ}_{E}\left(\theta_{0}^{\left(1\right)},{\tilde{\theta }}_{EL}^{\left(2\right)}\right)\overset{d}{\to }\chi^{2}\left(q\right).$$

### 3.4. Conditional Maximum Likelihood Estimation

It is straightforward to derive the log-likelihood function logL(θ) from the one-step-ahead transition probability (6) of model (4). In time-series models, the probability distribution of the first observation $Y_{1}$ is unknown, and its influence on the likelihood function is minimal when the sample size T is sufficiently large. Thus, we focus only on the conditional likelihood function. Given that the log-conditional likelihood function is a nonlinear function of the parameter vector θ=(β,λ), we employ numerical methods to solve:

$${\hat{\theta }}_{CML}=argmin_{\theta }\left(logL\left(\theta \right)\right).$$

To obtain the asymptotic distribution of ${\hat{\theta }}_{CML}$, we need to verify the regularity conditions presented in Billingsley [22]. The satisfaction of these conditions can be directly observed from the model-building process in Section 2 and the assumptions provided in Section 3. Therefore, the proof is omitted. We arrive at the following theorem:

**Theorem** **4.** *Under assumptions (A1)–(A6), the conditional maximum likelihood estimator* ${\hat{\theta }}_{CML}$ *consistently estimates the true parameter* $\theta_{0}$ *and exhibits an asymptotic distribution:*

$$\sqrt{T}\left({\hat{\theta }}_{CML}-\theta_{0}\right)\overset{d}{\to }N\left(0,E^{-1}\right),$$

*where* $E=E\left(\frac{𝜕\log\left(\mathbb{P}\left(X_{1}|X_{0}\right)\right)}{𝜕\theta }\cdot \frac{𝜕\log\left(\mathbb{P}\left(X_{1}|X_{0}\right)\right)}{𝜕\theta^{\prime }}\right)$ *represents the Fisher information matrix.*

**Remark** **9.** *Achieving CML estimation requires making specific assumptions about the probability distribution of* $Z_{t}$*. In this paper, we assume* $Z_{t}$ *follows a Poisson distribution with parameter* λ*. This strong assumption can result in significant errors or even inconsistency in statistical inference based on the CML method if the assumed model does not represent the true data-generating process. This constitutes the primary drawback of CML estimation. The impact of model mis-specification on CML estimation will be examined in the following numerical simulation section.*

## 4. Numerical Simulation

In this section, we set the function ν as a linear function, considering the following data-generating process:

(10) $$Y_{t}=\phi_{t}⊖Y_{t-1}+Z_{t},$$

(11) $$E\left(\phi_{t}|Y_{t}\right)={\frac{\exp\left(\beta_{0}+\beta_{1}Y_{t-1}\right)}{1+\exp\left(\beta_{0}+\beta_{1}Y_{t-1}\right)}}_{.}$$

Here, $\left\{Z_{t}\right\}$ represents an independently and identically distributed Poisson random variable sequence with a mean of λ. In subsequent numerical simulation studies, we mainly concentrate on three aspects: parameter estimation, interval estimation, and empirical likelihood ratio testing. All numerical simulations are conducted based on 1000 repeated sampling.

### 4.1. Parameter Estimation

We generate data using the above model and apply the CLS and CML methods to estimate parameters. Moreover, we define three statistical measures for evaluating estimation performance (using λ as an example):

$$\mathrm{Sample}\;\mathrm{bias}:\;\mathrm{Bias}=\bar{\lambda }-\lambda ,$$

$$\mathrm{Root}\;\mathrm{mean}\;\mathrm{square}\;\mathrm{error}:\;\mathrm{RMSE}=\sqrt{\frac{1}{1000}\sum_{i=1}^{1000}{\left({\hat{\lambda }}_{i}-\lambda \right)}^{2}},$$

$$\mathrm{Mean}\;\mathrm{absolute}\;\mathrm{percentage}\;\mathrm{error}:\;\mathrm{MAPE}=\frac{1}{1000}\sum_{i=1}^{1000}\left|\frac{{\hat{\lambda }}_{i}-\lambda }{\lambda }\right|.$$

In CML estimation, the score function is defined as:

$$\overset{T}{\underset{t=1}{\sum }}\frac{\left(\frac{𝜕}{𝜕\theta }\right)\left\{\int^{\;}\sum_{k=0}^{y_{t}}\frac{{\left(\phi y_{t-1}\right)}^{k}}{k!}\exp\left(-\phi y_{t-1}\right)f_{z}\left(y_{t}-k\right)\mathbb{P}\left(\phi_{t}=\phi |Y_{t-1}=y_{t-1}\right)d\phi \right\}}{\int^{\;}\sum_{k=0}^{y_{t}}\frac{{\left(\phi y_{t-1}\right)}^{k}}{k!}\exp\left(-\phi y_{t-1}\right)f_{z}\left(y_{t}-k\right)\mathbb{P}\left(\phi_{t}=\phi |Y_{t-1}=y_{t-1}\right)d\phi }=0.$$

In the CML estimation, we primarily consider four distribution cases for $\phi_{t}|Y_{t-1}$ when $Z_{t}$ follows a Poisson distribution. Let the variable $A_{t}=\frac{\exp\left(\beta_{0}+\beta_{1}Y_{t-1}\right)}{1+\exp\left(\beta_{0}+\beta_{1}Y_{t-1}\right)}$, and the function $dpois\left(x,l\right)=\frac{l^{x}}{x!}\exp\left(-l\right),l\geq 0,x\in \mathbb{N}$. Then:

(i) $\phi_{t}|Y_{t-1}$ is fixed at $A_{t}$, without any randomness. In this case, the log-likelihood function is:
  $$logL\left(\theta \right)=-\overset{T}{\underset{t=2}{\sum }}\log{\left(\overset{y_{t}}{\underset{k=0}{\sum }}\left(dpois\left(k,y_{t-1}A_{t}\right)\cdot dpois\left(y_{t}-k,\lambda \right)\right)\right)}_{.}$$
(ii) $\phi_{t}|Y_{t-1}$ follows a uniform distribution with mean $A_{t}$, minimum value 0, and maximum value $2A_{t}$. In this case, the log-likelihood function is:
  $$logL\left(\theta \right)=-\overset{T}{\underset{t=2}{\sum }}\log{\left(\overset{y_{t}}{\underset{k=0}{\sum }}\frac{dpois\left(y_{t}-k,\lambda \right)}{2k!y_{t-1}A_{t}}\cdot \left(\Gamma \left(k+1,0\right)-\Gamma \left(k+1,2A_{t}\right)\right)\right)}_{.}$$
  where $\Gamma \left(\alpha ,x\right)=\int_{x}^{\infty }t^{\alpha -1}\exp\left(-t\right)dt$.
(iii) $\phi_{t}|Y_{t-1}$ follows an exponential distribution with mean $A_{t}$. In this case, the log-likelihood function is:
  $$logL\left(\theta \right)=-\overset{T}{\underset{t=2}{\sum }}\log{\left(\overset{y_{t}}{\underset{k=0}{\sum }}\frac{A_{t}}{{\left(A_{t}+y_{t-1}\right)}^{k+1}}\cdot y_{t-1}^{k}\cdot dpois\left(y_{t}-k,\lambda \right)\right)}_{.}$$
(iv) $\phi_{t}|Y_{t-1}$ follows a chi-square distribution with the mean $A_{t}$. Specifically, the density function of $\phi_{t}|Y_{t-1}$ is:
  $$\mathbb{P}\left(\phi_{t}=\phi |Y_{t-1}=y_{t-1}\right)=\frac{1}{2^{A_{t}}\Gamma \left(A_{t}/2\right)}\phi^{\frac{A_{t}}{2}-1}\exp{\left(-\frac{\phi }{2}\right)}_{.}$$

Although $A_{t}$ is not an integer, we still call it a chi-square distribution. In this case, the log-likelihood function is:

$$logL\left(\theta \right)=-\overset{T}{\underset{t=2}{\sum }}\log{\left(\overset{y_{t}}{\underset{k=0}{\sum }}\frac{y_{t-1}}{k!}\cdot dpois\left(y_{t}-k,\;\lambda \right)\cdot \frac{1}{2^{\frac{A_{t}}{2}}\Gamma \left(\frac{A_{t}}{2},0\right)}\cdot \frac{\Gamma \left(\frac{A_{t}+2k}{2},0\right)}{{\left(\frac{1}{2}+y_{t-1}\right)}^{\frac{A_{t}+2k}{2}}}\right)}_{\;\;.}$$

The specific simulation results are shown in the table below:

From Table 1, we can observe that for both CLS and CML estimators, as the sample size T gradually increases, BIAS, RMSE, and MADE all decline, indicating the consistency of these estimators. Notably, both CLS and CML yield satisfactory parameter estimates. In large samples, CLS and CML estimates are approximately equal, while in small samples, under the premise of a correctly specified model, CML tends to provide superior estimation precision. Furthermore, we present an additional set of parameter estimation simulation results in the Appendix A, as shown in Table A1.

**Figure 1:**
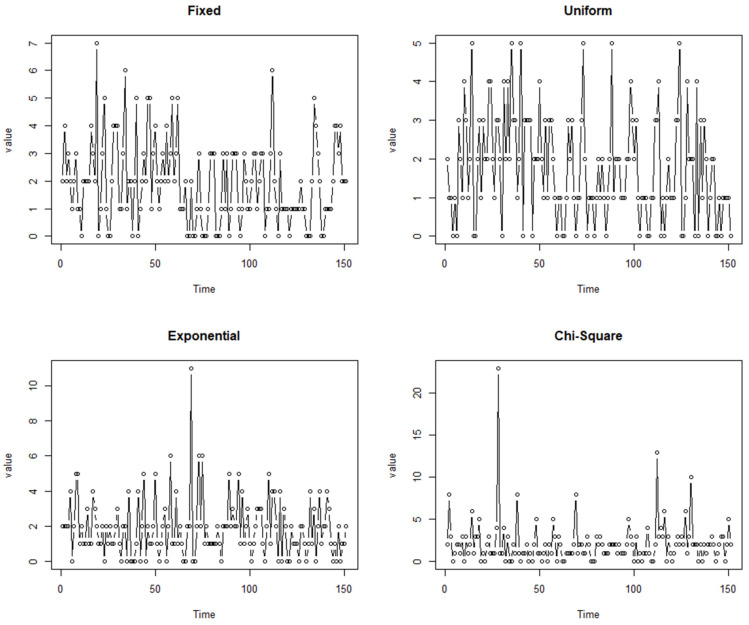
Typical trajectory of the model with $\beta_{0}=1$, $\beta_{1}=-0.6$, and λ=1.2.

Figure 1 showcases the typical trajectory of data generated by models (10) and (11) with parameters $\beta_{0}=1$, $\beta_{1}=-0.6$, and λ=1.2. In this figure, “fixed” represents $\phi_{t}|y_{t-1}$ as a fixed parameter given $y_{t-1}$, “uniform” denotes $\phi_{t}|y_{t-1}$ following a uniform distribution, “exponential” signifies $\phi_{t}|y_{t-1}$ following an exponential distribution, and “chi-square” indicates $\phi_{t}|y_{t-1}$ following a chi-square distribution. Figure 1 reveals that some extreme values are present in the sample paths when $\phi_{t}|y_{t-1}$ follows either an exponential or chi-square distribution, with the latter capable of generating even higher extreme values. This suggests that these two distribution settings for $\phi_{t}|y_{t-1}$ contain a certain descriptive ability concerning the extreme values in the data.

As pointed out in Section 3.4, the CML method depends upon correct model specification. To evaluate the effects of model misspecification on parameter estimation, we consider $\left\{Z_{t}\right\}$ as an independently and identically distributed geometric random-variable sequence with a mean of λ within the data generation process (10) and (11). Subsequently, we employ both the CLS and CML methods for estimation, presenting the results in the table below.

From Table 2, we can observe that the three statistical measures BIAS, RMSE, and MAPE for the CML estimator have noticeably increased compared to the CLS estimator. This indicates that model misspecification significantly impacts CML estimation, necessitating appropriate model selection efforts before employing the CML estimation method. As long as the conditional expectation $E\left(Y_{t}|Y_{t-1}\right)$ is correctly specified, CLS estimation will be more robust than CML estimation. Moreover, we provide the parameter estimation simulation results obtained under the misspecification of the $\phi_{t}|y_{t-1}$ distribution in the Appendix A, as shown in Table A2.

### 4.2. Interval Estimation

We perform a numerical simulation study on the coverage frequency of the interval estimation, as proposed in Theorem 2 and Remark 5, for the true values in the model. We consider parameter settings of $\beta_{0}=1$, $\beta_{1}=-0.6$, and λ=1.2. The nominal levels considered are 0.90 and 0.95, with the specific simulation results presented in the following table:

From Table 3, we can observe that as the sample size T increases, the coverage frequency of interval estimation gradually approaches the nominal level. Even with smaller sample sizes, the coverage frequency of the interval estimation for the true values remains satisfactory. This result suggests that the data-driven interval estimation has achieved commendable performance.

### 4.3. Empirical Likelihood Test

Lastly, we perform a numerical simulation study on the empirical likelihood test (EL test). For the observation-driven parameter model defined by data generation processes (10) and (11), we aim to test whether $\beta_{1}$ equals 0. If $\beta_{1}=0$, our model’s parameters are not driven by observations. We employ models (10) and (11) to generate sequences, assuming $\phi_{t}|y_{t-1}$ is a fixed parameter, and perform estimation under the null hypothesis. Then, we compare the test statistic proposed in Theorem 3 with the upper 0.90 and 0.95 quantiles of the corresponding chi-square distribution; if the EL test statistic exceeds the critical value, we reject the null hypothesis.

Initially, we investigate scenarios in which the true value of $\beta_{1}$ for the data generation process equals 0, considering the following hypotheses:

$${ℍ}_{0}:\beta_{1}=b\neq 0\;\;{ℍ}_{1}:\beta_{1}\neq b.$$

where b is a nonnegative constant, the simulation results for the test power are presented below (the simulation results for ${ℍ}_{0}:\beta_{1}=0$ represent the frequency of Type I errors).

Next, we examine the scenarios where the true value of $\beta_{1}$ in the data generation process is not equal to 0, considering the following hypotheses:

$${ℍ}_{0}:\beta_{1}=0\;\;{ℍ}_{1}:\beta_{1}\neq 0.$$

The simulation results for the test power are as follows.

From Table 4 and Table 5, we observe that the Type I error frequency of the EL test gradually diminishes to the corresponding confidence level as the sample size T increases, while the test power concurrently ascends to 1. Notably, in small sample scenarios, when the true value of $\beta_{1}$ is 0, the test power level for ${ℍ}_{0}:\beta_{1}=-0.1$ is relatively low. Likewise, when the true value of $\beta_{1}$ is −0.1, the test power for ${ℍ}_{0}:\beta_{1}=0$ exhibits a similar pattern. Overall, however, the EL test performs satisfactorily when the gap between the true and hypothesized values of $\beta_{1}$ is relatively large, or in cases involving large samples. Owing to space constraints, we include in the Appendix A, the EL test simulation results for the parameter λ under $\phi_{t}|y_{t-1}$ following four distinct random distributions, as shown in Table A3.

It is crucial to note that the estimation equation employed in the empirical likelihood test solely reflects the linear mean structure inherent in the data-generating process. For more intricate and nonlinear coefficient random distributions, the test exhibits limited descriptive capacity. As a result, we advise against utilizing the empirical likelihood test in cases where $\phi_{t}|y_{t-1}$ is stochastic. In Appendix A, we present numerical simulation results pertaining to the empirical likelihood test when $\phi_{t}|y_{t-1}$ adheres to an exponential distribution. As evidenced by Table A4, the empirical likelihood test demonstrates a very high frequency of Type I errors when $\phi_{t}|y_{t-1}$ conforms to an exponential distribution. Consequently, we discourage the use of the empirical likelihood test in such circumstances.

## 5. Real Data Application

In this section, we analyze the daily download count data for the software CWB TeXpert, covering the period from 1 June 2006, to 28 February 2007, resulting in a sample size of T = 267. This dataset is made available on the Supplementary webpage associated with Weiß [23].

**Figure 2:**
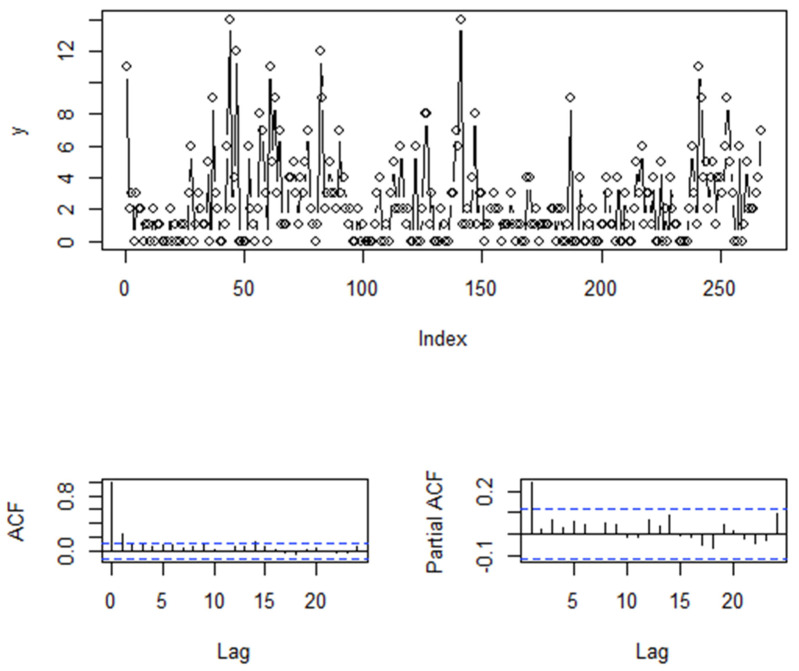
Sample Path of Software Download Data and Corresponding ACF and PACF Plots.

From the sample path in Figure 2, we observe that this data contains a considerable number of extreme values. Simultaneously, the ACF and PACF plots suggest that the sample might have originated from a first-order autoregressive data-generating process. We proceed to analyze this data using the models introduced in this paper. For the CML estimation, $CML_{fix}$ in the table below represents $\phi_{t}|y_{t-1}$ as a fixed parameter, $CML_{unif}$ denotes $\phi_{t}|y_{t-1}$ following a uniform distribution, $CML_{exp}$ signifies $\phi_{t}|y_{t-1}$ following an exponential distribution, and $CML_{chi}$ indicates $\phi_{t}|y_{t-1}$ following a chi-square distribution. Additionally, for comparison purposes, we applied the model proposed by Yu et al. [18] to this dataset, which is denoted as $CML_{geom}$ in the subsequent table:

The estimation results are displayed in Table 6, where we provide AIC and BIC values for the four distributions that $\phi_{t}|y_{t-1}$ may follow. Based on these two information criteria, we show a preference for models in which $\phi_{t}|y_{t-1}$ follows either a chi-square distribution or an exponential distribution. This preference might be attributable to the presence of extreme values in the sample path, as anticipated. As observed in Figure 1 in Section 4, models with $\phi_{t}|y_{t-1}$ following either a chi-square or exponential distribution prove more effective in capturing data characterized by extreme values.

## 6. Discussion and Conclusions

In this paper, we propose a first-order integer-valued autoregressive time series model based on the Poisson thinning operator. The parameters of this model are observation-driven and may follow specific random distributions, resulting in time-varying autoregressive coefficients. We established the ergodicity of this model and performed estimation and hypothesis testing using conditional least squares (CLS), conditional maximum likelihood (CML), and empirical likelihood (EL) methods. Additionally, we provided a data-driven interval estimation.

In the numerical simulation study, we compared the parameter estimation performance of CLS and CML, verified the coverage frequency of the interval estimation for the true parameter values in the data generation process, and conducted corresponding simulation studies for the EL test. The simulation study reveals that the properties of the CML estimation depend on the correct model specification, while the CLS estimation demonstrates a degree of robustness against model misspecifications.

**Figure A1:**
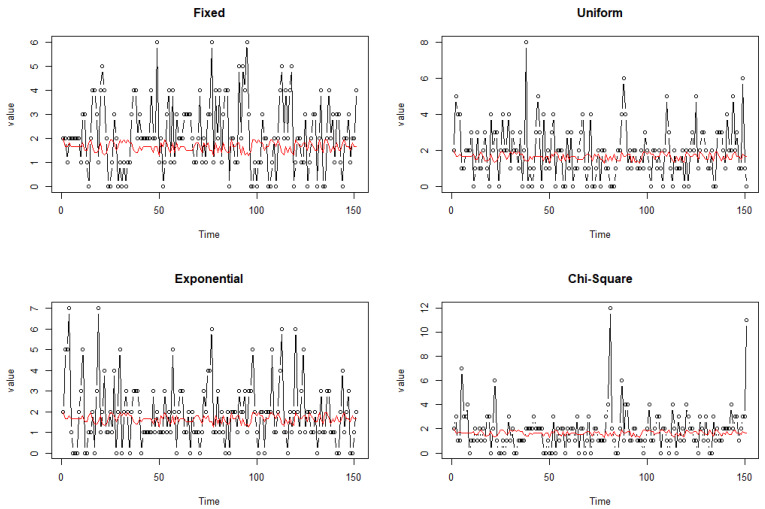
The black line represents the sample trajectory, and the red line denotes the one-step-ahead forecast trajectory. Parameter: $\beta_{0}=1$, $\beta_{1}=-0.6$, λ=1.2.

In future research, observation-driven parameter integer-valued time series models offer numerous promising avenues for development. In this discussion, a brief overview of some of these directions is provided:

(1) Combining observation-driven parameters with self-driven parameters, namely self-exciting threshold models: the SETINAR model proposed by Montriro, Scotto, and Pereira [16] is defined as follows:
  (12) $$\begin{array}{c} Y_{t}=\{\begin{array}{c} \overset{p^{\left(1\right)}}{\underset{i=1}{\sum }}\alpha_{i}^{\left(1\right)}\circ Y_{t-i}+Z_{t}^{\left(1\right)},\;Y_{t-d}\leq R,\\ \overset{p^{\left(2\right)}}{\underset{i=1}{\sum }}\alpha_{i}^{\left(2\right)}\circ Y_{t-i}+Z_{t}^{\left(2\right)},\;Y_{t-d}>R, \end{array} \end{array}$$
  in this model, $p^{\left(1\right)}$ and $p^{\left(2\right)}$ represent given positive integers, with $\overset{p^{\left(j\right)}}{\underset{i=1}{\sum }}\alpha_{i}^{\left(j\right)}\in \left(0,1\right)$ for j=1,2. Additionally, the innovation series $\left\{Z_{t}^{\left(1\right)}\right\}$ and $\left\{Z_{t}^{\left(2\right)}\right\}$ possess probability distributions $F_{1}$ and $F_{2}$ on the set of natural numbers ${\mathbb{N}}_{0}$, respectively. The constant R represents the threshold value responsible for the structural transition in the lagged d-period observation excitation model. Montriro, Scotto, and Pereira [16] demonstrated that model 6.1 possesses a strictly stationary solution when $p^{\left(1\right)}=p^{\left(2\right)}=1$. By effectively combining observation-driven parameter models with self-driven parameter models and flexibly selecting thinning operators, a more diverse range of integer-valued time series models can be characterized.
(2) Expanding upon current observation-driven models to incorporate higher-order models: Du and Li [24] introduced the INAR(p) model:
  (13) $$\begin{array}{c} Y_{t}=\alpha_{1}\circ Y_{t-1}+\cdots +\alpha_{p}\circ Y_{t-p}+Z_{t}, \end{array}$$
  in this model, $\overset{p}{\underset{i=1}{\sum }}\alpha_{i}<1$, and $\left\{Z_{t}\right\}$ represents a sequence of integer-valued random variables defined on the set of natural numbers ${\mathbb{N}}_{0}$. Existing observation-driven models are primarily first-order models. By extending these models to higher-order versions, the capability to describe more intricate and complex parameter dynamics can be achieved. It is important to note that when progressing to higher-order models, the technique utilized in the proof of Property 2. is no longer applicable for establishing the model’s ergodicity. As a result, new proof methods need to be sought from related Markov chain theories.
(3) Extending the observation-driven parameter setting to Integer-valued Autoregressive Conditional Heteroskedasticity (INARCH) models: Fokianos, Rahbek, and Tjøstheim [25] proposed the INARCH model (which they referred to as Poisson Autoregressive) as follows:
  (14) $$\begin{array}{c} \begin{array}{c} Y_{t}|{ℱ}_{t-1}\sim Poisson\left(\lambda_{t}\right),\\ \lambda_{t}=d+\alpha \lambda_{t-1}+\beta Y_{t-1}, \end{array} \end{array}$$
  where α≥0, β≥0, and α+β<1. This model is a natural extension of the generalized linear model and helps to capture the fluctuating changes of observed variables over time. Another advantage of this model is its simplicity, which makes it easy to establish the likelihood function of the INARCH model. Extending the observation-driven parameter setting to integer-valued autoregressive conditional heteroskedasticity models allows the model to describe the driving effect of the fluctuations of observed variables on the parameters. However, the challenge in doing so lies in the fact that, compared to the INAR model used in this paper, the ergodicity of the INARCH model is more difficult to establish.
(4) Forecasting Integer-Valued Time Series: In time series research, it is common to employ h-step forward conditional expectations for forecasting:
  (15) $$\begin{array}{c} {\hat{Y}}_{t+h}=E\left(Y_{t+h}|Y_{t}\right) \end{array}$$
  Nonetheless, this approach does not guarantee that the predicted values will be integers, and such predictions primarily describe the expected characteristics of the model, without capturing potentially time-varying coefficients or other features, as illustrated in Figure A1. Furthermore, Freeland and McCabe [26] highlighted that utilizing conditional medians or conditional modes for forecasting could be misleading. Consequently, it is essential to adopt innovative forecasting methods for integer-valued time series analysis. The rapid advancement of machine learning and deep learning in recent years has offered numerous new perspectives, such as the deep autoregressive model based on autoregressive recurrent neural network proposed by Salinas, Flunkert, Gasthaus, and Januschowski [10], which may hold significant potential for widespread application in the domain of integer-valued time series.

## Figures and Tables

**Table 1:** Parameter Estimation Simulation Results.

Sample Size	$\beta_{0}^{\left(CLS\right)}$	$\beta_{0}^{\left(CML\right)}$	$\beta_{1}^{\left(CLS\right)}$	$\beta_{1}^{\left(CML\right)}$	$\lambda^{\left(CLS\right)}$	$\lambda^{\left(CML\right)}$
$\mathrm{Parameter}:\;\beta_{0}=1$$,\;\beta_{1}=-0.6$, λ=1.2 $\phi_{t}|y_{t-1}$ is fixed.						
T = 300						
BIAS	0.0571	0.0471	−0.0321	−0.0287	0.0051	0.0059
RMSE	0.7399	0.6983	0.2096	0.2008	0.1368	0.1337
MAPE	0.5636	0.5486	0.2691	0.2619	0.0909	0.0886
T = 500						
BIAS	0.0506	0.0407	−0.0251	−0.0221	0.0033	0.0042
RMSE	0.5678	0.5562	0.1556	0.1523	0.1113	0.1091
MAPE	0.4443	0.4346	0.1978	0.1946	0.0738	0.0721
T = 800						
BIAS	0.0349	0.0246	−0.0152	−0.0127	−0.0011	0.0004
RMSE	0.4165	0.4076	0.1188	0.1163	0.0828	0.0817
MAPE	0.3327	0.3254	0.1587	0.1554	0.0546	0.0535
T = 1200						
BIAS	0.0139	0.0071	−0.0074	−0.0055	0.0009	0.0017
RMSE	0.3471	0.3393	0.0951	0.0931	0.0697	0.0688
MAPE	0.2726	0.2686	0.1252	0.1234	0.0465	0.0459
T = 2000						
BIAS	0.0112	0.0085	−0.0058	−0.0053	0.0017	0.0023
RMSE	0.2719	0.2711	0.0732	0.0728	0.0533	0.0525
MAPE	0.2195	0.2176	0.0981	0.0978	0.0352	0.0347
$\mathrm{Parameter}:\;\beta_{0}=1$$,\;\beta_{1}=-0.6$, λ=1.2 $\phi_{t}|y_{t-1}$ follows a uniform distribution.						
T = 300						
BIAS	0.0865	0.0428	−0.0456	−0.0354	0.0046	0.0121
RMSE	0.8065	0.7395	0.2301	0.2163	0.1454	0.1361
MAPE	0.6267	0.5773	0.2865	0.2696	0.0964	0.0903
T = 500						
BIAS	0.0312	0.0076	−0.0228	−0.0169	0.0043	0.0082
RMSE	0.5636	0.5288	0.1567	0.1488	0.1052	0.0997
MAPE	0.4493	0.4239	0.2046	0.1968	0.0703	0.0657
T = 800						
BIAS	0.0292	0.0062	−0.0165	−0.0113	0.0038	0.0079
RMSE	0.4503	0.4233	0.1244	0.1191	0.0852	0.0793
MAPE	0.3587	0.3373	0.1651	0.1575	0.0563	0.0525
T = 1200						
BIAS	0.0249	0.0133	−0.0127	−0.0108	0.0003	0.0031
RMSE	0.3513	0.3295	0.0971	0.0923	0.0689	0.0639
MAPE	0.2815	0.2627	0.1289	0.1249	0.0464	0.0428
T = 2000						
BIAS	0.0062	−0.0019	−0.0041	−0.0023	0.0016	0.0032
RMSE	0.2735	0.2529	0.0749	0.0719	0.0529	0.0483
MAPE	0.2165	0.1997	0.0983	0.0942	0.0353	0.0323
$\mathrm{Parameter}:\;\beta_{0}=1$$,\;\beta_{1}=-0.6$, λ=1.2 $\phi_{t}|y_{t-1}$ follows an exponential distribution.						
T = 300						
BIAS	0.1165	0.0594	−0.0594	−0.0491	0.0048	0.0135
RMSE	0.8356	0.7986	0.2648	0.2541	0.1407	0.1138
MAPE	0.6249	0.5392	0.3071	0.2785	0.0931	0.0752
T = 500						
BIAS	0.0174	−0.0175	−0.0195	−0.0116	0.0019	0.0088
RMSE	0.5929	0.5009	0.1649	0.1507	0.1059	0.0871
MAPE	0.4677	0.3955	0.2133	0.1932	0.0701	0.0582
T = 800						
BIAS	0.0389	0.0125	−0.0177	−0.0119	−0.0008	0.0042
RMSE	0.4646	0.3871	0.1267	0.1149	0.0839	0.0657
MAPE	0.3673	0.3052	0.1644	0.1486	0.0563	0.0438
T = 1200						
BIAS	0.0236	0.0014	−0.0103	−0.0057	0.0016	0.0057
RMSE	0.3709	0.3109	0.0997	0.0903	0.0687	0.0542
MAPE	0.2879	0.2472	0.1299	0.1201	0.0451	0.0362
T = 2000						
BIAS	0.0196	0.0074	−0.0091	−0.0072	−0.0021	0.0009
RMSE	0.2837	0.2493	0.0795	0.0746	0.0527	0.0427
MAPE	0.2261	0.1991	0.1047	0.0983	0.0356	0.0286
$\mathrm{Parameter}:\;\beta_{0}=1$$,\;\beta_{1}=-0.6$, λ=1.2 $\phi_{t}|y_{t-1}$ follows a chi−square distribution.						
T = 300						
BIAS	0.9382	0.2286	−0.3652	−0.1152	−0.0292	0.0041
RMSE	3.7397	1.2307	1.7201	0.5974	0.1471	0.0955
MAPE	1.3992	0.7657	0.8326	0.4475	0.0945	0.0636
T = 500						
BIAS	0.3437	0.1486	−0.1325	−0.0738	−0.0213	0.0007
RMSE	1.0769	0.7791	0.3808	0.2767	0.1129	0.0737
MAPE	0.7455	0.5794	0.4262	0.3345	0.0738	0.0493
T = 800						
BIAS	0.1769	0.0771	−0.0628	−0.0339	−0.0139	−0.0006
RMSE	0.7215	0.5257	0.2459	0.1844	0.0889	0.0556
MAPE	0.5301	0.4118	0.2954	0.2363	0.0586	0.0374
T = 1200						
BIAS	0.0883	0.0452	−0.0322	−0.0216	−0.0054	0.0012
RMSE	0.5649	0.4368	0.1849	0.1498	0.0703	0.0455
MAPE	0.4353	0.3445	0.2367	0.1949	0.0469	0.0299
T = 2000						
BIAS	0.0766	0.0269	−0.0292	−0.0128	−0.0057	0.0005
RMSE	0.4163	0.3267	0.1345	0.1103	0.0542	0.0371
MAPE	0.3256	0.2585	0.1706	0.1441	0.0361	0.0246

**Table 2:** Parameter Estimation Simulation Results under Model Misspecification.

Sample Size	$\beta_{0}^{\left(CLS\right)}$	$\beta_{0}^{\left(CML\right)}$	$\beta_{1}^{\left(CLS\right)}$	$\beta_{1}^{\left(CML\right)}$	$\lambda^{\left(CLS\right)}$	$\lambda^{\left(CML\right)}$
$\mathrm{Parameter}:\;\beta_{0}=1$$,\;\beta_{1}=-0.6$, λ=1.2 $\phi_{t}|y_{t-1}$ follows a uniform distribution, $Z_{t}$ follows a geometric distribution.						
T = 300						
BIAS	0.1823	0.8261	−0.1275	−0.1563	−0.0057	−0.1187
RMSE	1.2279	1.5387	0.7587	0.4665	0.1577	0.1798
MAPE	0.8237	1.0551	0.4661	0.4531	0.1027	0.1257
T = 500						
BIAS	0.0931	0.7121	−0.0632	−0.1079	−0.0009	−0.1198
RMSE	0.7686	1.0457	0.4375	0.2613	0.1198	0.1574
MAPE	0.5752	0.8394	0.3016	0.3169	0.0786	0.1118
T = 800						
BIAS	0.0858	0.6913	−0.0346	−0.0914	0.0001	−0.1199
RMSE	0.5812	0.9088	0.1651	0.1954	0.1006	0.1468
MAPE	0.4509	0.7538	0.2049	0.2451	0.0657	0.1069
T = 1200						
BIAS	0.0193	0.6389	−0.0132	−0.0732	0.0043	−0.01191
RMSE	0.4427	0.7848	0.1234	0.1503	0.0829	0.1385
MAPE	0.3495	0.6687	0.1607	0.1913	0.0545	0.1027
T = 2000						
BIAS	0.0224	0.6386	−0.0116	−0.0711	0.0021	−0.1213
RMSE	0.3576	0.7362	0.0951	0.1243	0.0625	0.1321
MAPE	0.2796	0.6517	0.1234	0.1612	0.0416	0.1016

**Table 3:** Coverage Frequency of Interval Estimation.

$\mathrm{Parameter}:\;\beta_{0}=1$, $\beta_{1}=-0.6$, λ=1.2 $\phi_{t}|y_{t-1}$ is fixed.					
T	300	500	800	1200	2000
0.95	0.941	0.957	0.957	0.953	0.956
0.9	0.897	0.908	0.912	0.908	0.905
$\mathrm{Parameter}:\;\beta_{0}=1$, $\beta_{1}=-0.6$, λ=1.2 $\phi_{t}|y_{t-1}$ follows a uniform distribution.					
T	300	500	800	1200	2000
0.95	0.949	0.959	0.961	0.949	0.954
0.9	0.89	0.913	0.899	0.904	0.903
$\mathrm{Parameter}:\;\beta_{0}=1$, $\beta_{1}=-0.6$, λ=1.2 $\phi_{t}|y_{t-1}$ follows an exponential distribution.					
T	300	500	800	1200	2000
0.95	0.942	0.938	0.951	0.955	0.953
0.9	0.891	0.894	0.906	0.910	0.909
$\mathrm{Parameter}:\;\beta_{0}=1$, $\beta_{1}=-0.6$, λ=1.2 $\phi_{t}|y_{t-1}$ follows a chi−square distribution.					
T	300	500	800	1200	2000
0.95	0.905	0.917	0.918	0.92	0.939
0.9	0.854	0.853	0.856	0.864	0.881

**Table 4:** Empirical Likelihood Test for $\beta_{1}$ with a True Value of 0.

$\mathrm{Parameter}:\;\beta_{0}=1$, $\beta_{1}=0$, λ=1.2 $\phi_{t}|y_{t-1}$ is fixed, significance level 0.05.					
T	300	500	800	1200	2000
${ℍ}_{0}:\beta_{1}=0$ (true)	0.096	0.073	0.065	0.057	0.046
${ℍ}_{0}:\beta_{1}=-0.1$	0.296	0.386	0.658	0.823	0.935
${ℍ}_{0}:\beta_{1}=-0.2$	0.707	0.802	0.941	0.984	1
${ℍ}_{0}:\beta_{1}=-0.3$	0.778	0.837	0.988	1	1
${ℍ}_{0}:\beta_{1}=-0.4$	0.822	0.861	0.997	1	1
$\mathrm{Parameter}:\;\beta_{0}=1$, $\beta_{1}=0$, λ=1.2 $\phi_{t}|y_{t-1}$ is fixed, significance level 0.10.					
T	300	500	800	1200	2000
${ℍ}_{0}:\beta_{1}=0$ (true)	0.146	0.126	0.110	0.103	0.107
${ℍ}_{0}:\beta_{1}=-0.1$	0.399	0.447	0.716	0.874	0.976
${ℍ}_{0}:\beta_{1}=-0.2$	0.784	0.883	0.969	1	1
${ℍ}_{0}:\beta_{1}=-0.3$	0.823	0.904	0.993	1	1
${ℍ}_{0}:\beta_{1}=-0.4$	0.875	0.921	1	1	1

**Table 5:** Empirical Likelihood Test for $\beta_{1}$ with True Value Not Equal to 0.

$\mathrm{Parameter}:\;\beta_{0}=1,{ℍ}_{0}:\beta_{1}=0,\lambda =1.2$ $\phi_{t}|y_{t-1}$ is fixed, significance level 0.05.					
T	300	500	800	1200	2000
$\beta_{1}=-0.1$ (true)	0.363	0.536	0.608	0.751	0.907
$\beta_{1}=-0.2$ (true)	0.647	0.806	0.936	0.988	1
$\beta_{1}=-0.3$ (true)	0.768	0.935	1	1	1
$\beta_{1}=-0.4$ (true)	0.875	0.945	1	1	1
$\mathrm{Parameter}:\;\beta_{0}=1,{ℍ}_{0}:\beta_{1}=0,\lambda =1.2$ $\phi_{t}|y_{t-1}$ is fixed, significance level 0.10.					
T	300	500	800	1200	2000
$\beta_{1}=-0.1$ (true)	0.439	0.705	0.767	0.859	0.966
$\beta_{1}=-0.2$ (true)	0.751	0.877	0.96	1	1
$\beta_{1}=-0.3$ (true)	0.835	0.99	1	1	1
$\beta_{1}=-0.4$ (true)	0.941	0.997	1	1	1

**Table 6:** Model Estimation Results.

	CLS	$CML_{fix}$	$CML_{unif}$	$CML_{exp}$	$CML_{chi}$	$CML_{geom}$
$\beta_{0}$	0.302	0.209	1.379	1.305	0.658	1.244
$\beta_{1}$	−0.151	−0.143	−0.227	−0.244	−0.097	−0.231
λ	1.463	1.493	1.201	1.196	1.359	1.166
AIC	-	1243.986	1189.377	1151.465	1143.669	1184.96
BIC	-	1254.748	1200.138	1162.227	1154.431	1195.322

## Data Availability

The following supporting data can be downloaded at: http://www.wiley.com/go/weiss/discrete-valuedtimeseries, (accessed on 27 April 2023). The code has been uploaded as Supplementary File of this paper. Interested readers are also encouraged to request the relevant data and code from the authors directly through e-mail.

## Supplementary Materials

The following supporting information can be downloaded at: https://www.mdpi.com/article/10.3390/e25060859/s1.

## Appendix A

### Appendix A.1. Proofs

**Property** **A1.**

(i) *Given the data generation process (4), the following can be proved using the law of iterated expectation:*
  $$E\left(Y_{t}|Y_{t-1};\phi_{t}\right)=\phi_{t}Y_{t-1}+\lambda ,$$
  $$Var\left(Y_{t}|Y_{t-1};\phi_{t}\right)=\phi_{t}Y_{t-1}+\sigma_{Z}^{2}.$$
  *Using the formula* Var(Y)=Var(E(Y|X))+E(Var(Y|X))*, the result can be proved*
(ii) *By the law of iterated expectation, we know:*
  $$E\left(Y_{t}Y_{t-1}\right)=E\left(Y_{t-1}E\left(Y_{t}|Y_{t-1}\right)\right)=E\left(\phi_{t-1}Y_{t-1}^{2}+Y_{t-1}\lambda \right),$$
  $$E\left(Y_{t}\right)E\left(Y_{t-1}\right)=E\left(\frac{\exp\left[\nu \left(Y_{t-1};\beta \right)\right]}{1+\exp\left[\nu \left(Y_{t-1};\beta \right)\right]}Y_{t-1}+\lambda \right)E\left(Y_{t-1}\right).$$
  *From this, it follows that:*
  $$Cov\left(Y_{t},Y_{t-1}\right)=E\left(\frac{\exp\left[\nu \left(Y_{t-1};\beta \right)\right]}{1+\exp\left[\nu \left(Y_{t-1};\beta \right)\right]}Y_{t-1}^{2}\right)-E\left(\frac{\exp\left[\nu \left(Y_{t-1};\beta \right)\right]}{1+\exp\left[\nu \left(Y_{t-1};\beta \right)\right]}Y_{t-1}\right)E\left(Y_{t-1}\right).$$

**Property** **A2.** *According to Theorem 1 in Tweedie [27] (also see Meyn and Tweedie [28]), the sufficient condition for* $\left\{Y_{t}\right\}$ *to be an ergodic Markov chain is the existence of a set* K *and a measurable function* g *in the state space* 𝒴 *of* $\left\{Y_{t}\right\}$ *such that:*

$$\int_{𝒴}P\left(x,dy\right)g\left(y\right)\leq g\left(x\right)-1,\;x\in K^{c}.\;$$

*and for a constant B:*

$$\int_{𝒴}P\left(x,dy\right)g\left(y\right)=\lambda \left(x\right)\leq B<\infty ,\;x\in K.$$

*where* $P\left(x,A\right)=\mathbb{P}(Y_{t}\in A|Y_{t-1}=x)$. *The state space* 𝒴 *of* $\left\{Y_{t}\right\}$ *is the set of natural numbers* ℕ={0,1,2,3,…}*. Let* g(y)=y*, then we have:*

$$\int_{\mathbb{N}}P\left(x,dy\right)g\left(y\right)=\overset{\infty }{\underset{y=0}{\sum }}\mathbb{P}\left(Y_{t}=y|Y_{t-1}=x\right)=E\left(Y_{t}|Y_{t-1}=x\right)\;=\frac{\exp\left[\nu \left(Y_{t-1};\beta \right)\right]}{1+\exp\left[\nu \left(Y_{t-1};\beta \right)\right]}x+\lambda .\;$$

*Since* $\underset{y\in \mathbb{N}}{\sup}\nu \left(y;\beta \right)<\infty$*, then:*

$$\frac{\exp\left[\nu \left(Y_{t-1};\beta \right)\right]}{1+\exp\left[\nu \left(Y_{t-1};\beta \right)\right]}=\frac{1}{1+\exp\left[-\nu \left(x;\beta \right)\right]}\leq \frac{1}{1+\exp\left[-\underset{y\in \mathbb{N}}{\sup}\nu \left(y;\beta \right)\right]}<1.$$

*Therefore, we can choose a constant* 0<m<1, *such that* $\frac{\exp\left[\nu \left(Y_{t-1};\beta \right)\right]}{1+\exp\left[\nu \left(Y_{t-1};\beta \right)\right]}<m$*. Let* $N=\left|\frac{\lambda +1}{1-m}\right|+1$*, where* ⌊c⌋ *represents the floor function of* c*. Defining* K={0,1,2,…,N−1}*, we know:*

$$\int_{\mathbb{N}}P\left(x,dy\right)g\left(y\right)=E\left(Y_{t}|Y_{t-1}=x\right)<mx+\lambda <x-1=g\left(x\right)-1,\;x\in K^{c},\;$$

$$\int_{\mathbb{N}}P\left(x,dy\right)g\left(y\right)=E\left(Y_{t}|Y_{t-1}=x\right)<x+\lambda <N+\lambda <\infty ,\;x\in K.\;$$

*Hence, the data generation process* $\left\{Y_{t}\right\}$ *is ergodic.*

**Theorem** **A1.** *According to Theorems 5 and 6 in Klimko and Nelson [29], let* $g=E\left(Y_{t}|Y_{\left(t-1\right)}\right)$*, if the following four conditions hold, then Theorem 1 in this paper holds:*

(i) $\frac{𝜕g}{𝜕\theta_{i}}$, $\frac{{𝜕}^{2}g}{𝜕\theta_{i}𝜕\theta_{j}}$, $\frac{{𝜕}^{3}g}{𝜕\theta_{i}𝜕\theta_{j}𝜕\theta_{k}}$, 1≤i,j,k≤ℓ+1, exists and are continuous with respect to θ.
(ii) For 1≤i,j≤ℓ+1, $E\left|\left(Y_{t}-g\right)\frac{𝜕g}{𝜕\theta_{i}}\right|<\infty$, $E\left|\left(Y_{t}-g\right)\frac{{𝜕}^{2}g}{𝜕\theta_{i}𝜕\theta_{j}}\right|<\infty$, $E\left|\frac{𝜕g}{𝜕\theta_{i}}\frac{𝜕g}{𝜕\theta_{j}}\right|<\infty$.
(iii) For 1≤i,j,k≤ℓ+1, there exist functions:
  $$H^{\left(0\right)}\left(Y_{t-1},\ldots ,Y_{0}\right),\;H_{i}^{\left(1\right)}\left(Y_{t-1},\ldots ,Y_{0}\right),\;H_{ij}^{\left(2\right)}\left(Y_{t-1},\ldots ,Y_{0}\right),\;H_{ijk}^{\left(3\right)}\left(Y_{t-1},\ldots ,Y_{0}\right),\;$$
  *such that*
  $$\left|g\right|\leq H^{\left(0\right)},\;\left|\frac{𝜕g}{𝜕\theta_{i}}\right|\leq H_{i}^{\left(1\right)},\;\left|\frac{{𝜕}^{2}g}{𝜕\theta_{i}𝜕\theta_{j}}\right|\leq H_{ij}^{\left(2\right)},\;\left|\frac{{𝜕}^{3}g}{𝜕\theta_{i}𝜕\theta_{j}𝜕\theta_{k}}\right|\leq H_{ijk}^{\left(3\right)},\;\;E\left|Y_{t}\cdot H_{ijk}^{\left(3\right)}\left(Y_{t-1},\ldots ,Y_{0}\right)\right|<\infty ,\;\;H^{\left(0\right)}\left(Y_{t-1},\ldots ,Y_{0}\right)H_{ijk}^{\left(3\right)}\left(Y_{t-1},\ldots ,Y_{0}\right)<\infty ,\;\;E\left|H_{i}^{\left(1\right)}\left(Y_{t-1},\ldots ,Y_{0}\right)H_{ij}^{\left(2\right)}\left(Y_{t-1},\ldots ,Y_{0}\right)\right|<\infty .$$
(iv) $E\left(Y_{t}|Y_{t-1},\ldots ,Y_{0}\right)=E\left(Y_{t}|Y_{\left(t-1\right)}\right)$, a.e., t≥1, 
  $$E\left(u_{t}^{2}\left(\theta \right)\left|\frac{𝜕g}{𝜕\theta_{i}}\frac{𝜕g}{𝜕\theta_{j}}\right|\right)<\infty ,\;$$
  *where* $u_{t}\left(\theta \right)=Y_{t}-E(Y_{t}|Y_{t-1})$.

*For model (4),* $g\left(\theta \right)=\frac{\exp\left[\nu \left(Y_{t-1};\beta \right)\right]}{1+\exp\left[\nu \left(Y_{t-1};\beta \right)\right]}Y_{t-1}+\lambda$*, for* 1≤i,j,k≤ℓ*, we have:*

$$\left|g\left(\theta \right)\right|<Y_{t-1}+\lambda ,\;\left|\frac{𝜕g}{𝜕\theta_{\ell +1}}\right|=1,\;\left|\frac{𝜕g}{𝜕\theta_{i}}\right|<\left|\frac{𝜕\nu }{𝜕\beta_{i}}\right|Y_{t-1},\;\left|\frac{{𝜕}^{2}g}{𝜕\theta_{i}𝜕\theta_{j}}\right|<\left(\left|\frac{𝜕g}{𝜕\theta_{i}}\frac{𝜕g}{𝜕\theta_{j}}\right|+\left|\frac{{𝜕}^{2}\nu }{𝜕\beta_{i}𝜕\beta_{j}}\right|\right)Y_{t-1},\;$$

$$\left|\frac{{𝜕}^{3}g}{𝜕\theta_{i}𝜕\theta_{j}𝜕\theta_{k}}\right|<\left(\left|\frac{𝜕\nu }{𝜕\beta_{i}}\frac{𝜕\nu }{𝜕\beta_{j}}\frac{𝜕\nu }{𝜕\beta_{k}}\right|+\left|\frac{{𝜕}^{2}\nu }{𝜕\beta_{i}𝜕\beta_{k}}\frac{𝜕\nu }{𝜕\beta_{j}}\right|+\left|\frac{{𝜕}^{2}\nu }{𝜕\beta_{j}𝜕\beta_{k}}\frac{𝜕\nu }{𝜕\beta_{i}}\right|+\left|\frac{{𝜕}^{2}\nu }{𝜕\beta_{i}𝜕\beta_{j}}\frac{𝜕\nu }{𝜕\beta_{k}}\right|\right)Y_{t-1}+\left|\frac{{𝜕}^{3}\nu }{𝜕\beta_{i}𝜕\beta_{j}𝜕\beta_{k}}\right|Y_{t-1}.\;$$

*Note that the second- and third-order partial derivatives of the function* g *with respect to* λ *are both 0. According to assumptions (A2) and (A3),* $\frac{𝜕g}{𝜕\theta_{i}}$*,* $\frac{{𝜕}^{2}g}{𝜕\theta_{i}𝜕\theta_{j}}$*), and* $\frac{{𝜕}^{3}g}{𝜕\theta_{i}𝜕\theta_{j}𝜕\theta_{k}}$*,* 1≤i,j,k≤ℓ+1*, exist and are continuous with respect to* θ*. According to assumption (A5),* $V\left(\theta_{0}\right)$ *is non-singular. Based on assumptions (A1), (A4), and the Hölder inequality, all four conditions are satisfied. Thus, Theorem 1 holds.*

**Lemma** **A1.** $\left\{M_{t}\left(\theta \right)M_{t}{\left(\theta \right)}^{\prime }\right\}$ *is an integrable process.*

Note that $\frac{\exp\left[\nu \left(Y_{t-1};\;\beta \right)\right]}{1+\exp\left[\nu \left(Y_{t-1};\;\beta \right)\right]}<1$, $\frac{1}{1+\exp\left[\nu \left(Y_{t-1};\;\beta \right)\right]}\leq 1$. According to assumption (A4), if i≤ℓ, then:

$$E\left(m_{ti}m_{ti}\right)\leq E\left\{{\left[Y_{t}-\frac{\exp\left[\nu \left(Y_{t-1};\beta \right)\right]}{1+\exp\left[\nu \left(Y_{t-1};\beta \right)\right]}Y_{t-1}-\lambda \right]}^{2}\frac{𝜕\nu \left(Y_{t-1};\beta \right)}{𝜕\beta_{i}}\frac{𝜕\nu \left(Y_{t-1};\beta \right)}{𝜕\beta_{i}}Y_{t-1}^{2}\right\}$$

$$\leq \sqrt{E{\left[Y_{t}-\frac{\exp\left[\nu \left(Y_{t-1};\beta \right)\right]}{1+\exp\left[\nu \left(Y_{t-1};\beta \right)\right]}Y_{t-1}-\lambda \right]}^{4}}\sqrt{E\left(N^{4}\left(y\right)Y_{t-1}^{4}\right)}$$

$$\leq \sqrt{E{\left[Y_{t}-\frac{\exp\left[\nu \left(Y_{t-1};\beta \right)\right]}{1+\exp\left[\nu \left(Y_{t-1};\beta \right)\right]}Y_{t-1}^{\;}-\lambda \right]}^{4}}\sqrt{\sqrt{E\left(N^{8}\left(y\right)\right)}\sqrt{EY_{t-1}^{8}}}<\infty .$$

Similarly, we can derive that:

If i,j≤ℓ, i≠j, then:

$$E\left(m_{ti}m_{tj}\right)\leq E\left\{{\left[Y_{t}-\frac{\exp\left[\nu \left(Y_{t-1};\beta \right)\right]}{1+\exp\left[\nu \left(Y_{t-1};\beta \right)\right]}Y_{t-1}-\lambda \right]}^{2}\frac{𝜕\nu \left(Y_{t-1};\beta \right)}{𝜕\beta_{i}}\frac{𝜕\nu \left(Y_{t-1};\beta \right)}{𝜕\beta_{j}}Y_{t-1}^{2}\right\}<\infty .$$

If i≤ℓ, j=ℓ+1, then:

$$E\left(m_{ti}m_{tj}\right)\leq E\left\{{\left[Y_{t}-\frac{\exp\left[\nu \left(Y_{t-1};\beta \right)\right]}{1+\exp\left[\nu \left(Y_{t-1};\beta \right)\right]}Y_{t-1}-\lambda \right]}^{2}\frac{𝜕\nu \left(Y_{t-1};\beta \right)}{𝜕\beta_{i}}Y_{t-1}\right\}<\infty .\;$$

If i=ℓ+1, then:

$$E\left(m_{ti}m_{ti}\right)\leq E\left\{{\left[Y_{t}-\frac{\exp\left[\nu \left(Y_{t-1};\beta \right)\right]}{1+\exp\left[\nu \left(Y_{t-1};\beta \right)\right]}Y_{t-1}-\lambda \right]}^{2}\right\}<\infty .$$

**Lemma** **A2.** $max_{1\leq t\leq T}\|M_{t}\left(\theta \right)\|=o_{p}\left(T^{\frac{1}{2}}\right)$.

Given assumption (A4) and Lemma A1, it follows that $E\left(M_{t}\left(\theta \right)\prime M_{t}\left(\theta \right)\right)<\infty$, resulting in $\overset{\infty }{\underset{t=1}{\sum }}\mathbb{P}\left(M_{t}\left(\theta \right)\prime M_{t}\left(\theta \right)\right)<\infty$. As the $\left\{Y_{t}\right\}$ series is strictly stationary, the event $\left\{\left\|M_{t}\left(\theta \right)\right\|>t^{\frac{1}{2}}\right\}$ occurs only a finite number of times with probability 1.

By a similar reasoning, let $M_{T}^{*}=max_{1\leq t\leq T}\|M_{t}\left(\theta )\right\|$, and for any ε>0, with probability 1, there will be only a finite number of T∈ℕ such that $M_{T}^{*}>\varepsilon \sqrt{T}$. Consequently:

$$limsup_{T}M_{T}^{*}T^{-\frac{1}{2}}\leq \varepsilon ,\;a.s.\;$$

This result implies that $M_{T}^{*}=o_{p}$.

**Lemma** **A3.** $max_{1\leq t\leq T}\frac{t}{\sum_{t=1}^{T}E\left(m_{ti}m_{tj}\right)}<\infty$, ∀1≤i,j≤ℓ+1.

The ergodicity property of $\left\{Y_{t}\right\}$ and Lemma A1 lead to:

$$max_{1\leq t\leq T}\frac{t}{\sum_{t=1}^{T}E\left(m_{ti}m_{tj}\right)}=max_{1\leq t\leq T}\frac{t}{T}{\left(E\left(m_{ti}m_{tj}\right)\right)}^{-1}\leq {\left(E\left(m_{ti}m_{tj}\right)\right)}^{-1}=O\left(1\right).$$

**Theorem** **A2.** *Given the ergodicity property of* $\left\{Y_{t}\right\}$ *and Lemma A1, and applying Theorem 14.6 from Davidson [30], we have:*

$$\frac{1}{T}\overset{T}{\underset{t=2}{\sum }}M_{t}\left(\theta_{0}\right)M_{t}{\left(\theta_{0}\right)}^{\prime }\overset{a.s.}{\to }E\left(M_{t}\left(\theta_{0}\right)M_{t}{\left(\theta_{0}\right)}^{\prime }\right).\;$$

*Let* ${ℱ}_{n}=\sigma \left(Y_{1},Y_{2},\ldots ,Y_{n}\right)$*,* ${\tilde{M}}_{ni}=\overset{n}{\underset{i=1}{\sum }}m_{ti}\left(\theta \right)$*,* 1≤i≤ℓ+1*. For* 1≤i≤ℓ*, we have*

$$E\left({\tilde{M}}_{ni}|{ℱ}_{n-1}\right)={\tilde{M}}_{\left(n-1\right)i},\;$$

$$+E\left(\left(Y_{n}-\frac{\exp\left[\nu \left(Y_{n-1};\beta \right)\right]}{1+\exp\left[\nu \left(Y_{n-1};\beta \right)\right]}Y_{n-1}-\lambda \right)\frac{\exp\left[\nu \left(Y_{n-1};\beta \right)\right]}{{\left(1+\exp\left[\nu \left(Y_{n-1};\beta \right)\right]\right)}^{2}}\frac{𝜕\nu \left(y;\beta \right)}{𝜕\beta_{i}}Y_{n-1}|{ℱ}_{n-1}\right),\;$$

$$={\tilde{M}}_{\left(n-1\right)i}.\;$$

*Similarly,* $E\left({\tilde{M}}_{n\left(\ell +1\right)}|{ℱ}_{n-1}\right)={\tilde{M}}_{\left(n-1\right)\left(\ell +1\right)}$*. Thus, for* 1≤i≤ℓ+1*,* $\left\{{\tilde{M}}_{ni},{ℱ}_{n},n\geq 0\right\}$ *is a martingale. Based on this and the ergodicity property of* $\left\{Y_{t}\right\}$*, and using Lemmas 2 and 3, applying Theorem 25.4 from Davidson* [30] *establishes that the conditions of Theorem 25.3 in Davidson [30] are satisfied, resulting in:*

$$\frac{1}{\sqrt{T}}\overset{T}{\underset{t=2}{\sum }}m_{ti}\left(\theta_{0}\right)\overset{d}{\to }N\left(0,E\left(m_{ti}^{2}\left(\theta \right)\right)\right).\;$$

*Furthermore, for any (*ℓ+1*)-dimensional vector* c≠0*, we have:*

$$\frac{1}{T}\overset{T}{\underset{t=2}{\sum }}c^{\prime }M_{t}\left(\theta_{0}\right)\overset{d}{\to }N\left(0,\sigma^{2}\right).\;$$

*Here,* $\sigma^{2}=E\left(c^{\prime }M_{t}\left(\theta_{0}\right)M_{t}{\left(\theta_{0}\right)}^{\prime }c\right)$*. Therefore, we have:*

$$\frac{1}{\sqrt{T}}\overset{T}{\underset{t=2}{\sum }}M_{t}\left(\theta_{0}\right)\overset{d}{\to }N\left(0,E\left(M_{t}\left(\theta_{0}\right)M_{t}{\left(\theta_{0}\right)}^{\prime }\right)\right).\;$$

*In summary,* $H\left(\theta_{0}\right)\overset{d}{\to }\chi^{2}\left(\ell +1\right)$.

**Lemma** **A4.** *Let* $\left\{Y_{t}\right\}$ *be an ergodic stationary random variable sequence; for any* i≥2*,* $E\left(Y_{t}|Y_{1},Y_{2},\ldots ,Y_{t-1}\right)=0$*,* a.s*., and* $E\left(Y_{1}^{2}\right)=1$*. Then:*

$$limsup\frac{\sum_{t=1}^{T}Y_{t}}{\sqrt{2TloglogT}}=1.$$

The proof can be found in Stout [31].

**Theorem** **A3.**
*Following steps similar to those in Yu, Wang, and Yang [18] and Qin and Lawless [20], we can show (by replacing the usage of the double logarithm law with Lemma A4):*

$$\gamma \left(\theta \right)=\left[\frac{1}{T}\overset{T}{\underset{t=2}{\sum }}M_{t}\left(\theta \right)M_{t}{\left(\theta \right)}^{\prime }\right]\frac{1}{T}\overset{T}{\underset{t=}{\sum }}M_{t}\left(\theta \right)+o\left(T^{\frac{1}{3}}\right).$$

$$2{ℒ}_{E}\left(\theta_{0}\right)={\left[\overset{T}{\underset{t=}{\sum }}M_{t}\left(\theta_{0}\right)\right]}^{\prime }{\left[\overset{T}{\underset{t=2}{\sum }}M_{t}\left(\theta_{0}\right)M_{t}{\left(\theta_{0}\right)}^{\prime }\right]}^{-1}\left[\overset{T}{\underset{t=}{\sum }}M_{t}\left(\theta_{0}\right)\right]+o\left(1\right).$$

*Furthermore:*

$$\sqrt{T}\left({\hat{\theta }}_{EL}-\theta_{0}\right)=S_{22}^{-1}S_{21}S_{11}^{-1}\frac{1}{\sqrt{T}}\overset{T}{\underset{t=}{\sum }}M_{t}\left(\theta_{0}\right)+o_{p}\left(1\right)\overset{d}{\to }N\left(0,S_{22}^{-1}\right),$$

$$2{ℒ}_{E}\left({\hat{\theta }}_{EL}\right)={\left[\overset{T}{\underset{t=}{\sum }}M_{t}\left(\theta_{0}\right)\right]}^{\prime }S_{3}\left[\overset{T}{\underset{t=}{\sum }}M_{t}\left(\theta_{0}\right)\right]+o_{p}\left(1\right),$$

*where:*

$$S_{3}=S_{11}^{-1}\left(I+S_{12}S_{22}^{-1}S_{21}S_{11}^{-1}\right),\;S_{22}^{-1}={\left[E\left(\frac{𝜕M_{t}\left(\theta_{0}\right)}{𝜕\theta^{\prime }}\right){\left(E\left(M_{t}\left(\theta_{0}\right)M_{t}{\left(\theta_{0}\right)}^{\prime }\right)\right)}^{-1}E\left(\frac{𝜕M_{t}\left(\theta_{0}\right)}{𝜕\theta }\right)\right]}^{-1},$$

$$S_{11}=E\left(M_{t}\left(\theta_{0}\right)M_{t}{\left(\theta_{0}\right)}^{\prime }\right),\;S_{12}=E\left(\frac{𝜕M_{t}\left(\theta_{0}\right)}{𝜕\theta^{\prime }}\right),\;S_{21}=S_{12}^{\prime }.$$

*Based on this, we perform a Taylor expansion of* $2{ℒ}_{E}\left(\theta_{0}^{\left(1\right)},{\tilde{\theta }}_{EL}^{\left(2\right)}\right)-2{ℒ}_{E}\left({\hat{\theta }}_{EL}^{\left(1\right)},{\hat{\theta }}_{EL}^{\left(2\right)}\right)$ *at* $\theta =\theta_{0}$*,* γ=0*:*

$$2{ℒ}_{E}\left(\theta_{0}^{\left(1\right)},{\tilde{\theta }}_{EL}^{\left(2\right)}\right)-2{ℒ}_{E}\left({\hat{\theta }}_{EL}^{\left(1\right)},{\hat{\theta }}_{EL}^{\left(2\right)}\right)\overset{d}{\to }$$

$${\left[E{\left(M_{t}\left(\theta_{0}\right)M_{t}{\left(\theta_{0}\right)}^{\prime }\right)}^{-\frac{1}{2}}\frac{1}{\sqrt{T}}\overset{T}{\underset{t=1}{\sum }}M_{t}\left(\theta_{0}\right)\right]}^{\prime }{\left(E\left(M_{t}\left(\theta_{0}\right)M_{t}{\left(\theta_{0}\right)}^{\prime }\right)\right)}^{-\frac{1}{2}}$$

$$\times \{E\left(\frac{𝜕M_{t}\left(\theta_{0}\right)}{𝜕\theta }\right){\left[E\left(\frac{𝜕M_{t}\left(\theta_{0}\right)}{𝜕\theta^{\prime }}\right){\left(E\left(M_{t}\left(\theta_{0}\right)M_{t}{\left(\theta_{0}\right)}^{\prime }\right)\right)}^{-1}E\left(\frac{𝜕M_{t}\left(\theta_{0}\right)}{𝜕\theta }\right)\right]}^{-1}E\left(\frac{𝜕M_{t}\left(\theta_{0}\right)}{𝜕\theta^{\prime }}\right)-$$

$$\left(\frac{𝜕M_{t}\left(\theta_{0}\right)}{𝜕\theta_{\left(1\right)}}\right){\left[E\left(\frac{𝜕M_{t}\left(\theta_{0}\right)}{𝜕\theta_{\left(1\right)}^{\prime }}\right){\left(E\left(M_{t}\left(\theta_{0}\right)M_{t}{\left(\theta_{0}\right)}^{\prime }\right)\right)}^{-1}E\left(\frac{𝜕M_{t}\left(\theta_{0}\right)}{𝜕\theta_{\left(1\right)}}\right)\right]}^{-1}E\left(\frac{𝜕M_{t}\left(\theta_{0}\right)}{𝜕\theta_{\left(1\right)}^{\prime }}\right)\}$$

$$\times {\left(E\left(M_{t}\left(\theta_{0}\right)M_{t}{\left(\theta_{0}\right)}^{\prime }\right)\right)}^{-\frac{1}{2}}\left[E{\left(M_{t}\left(\theta_{0}\right)M_{t}{\left(\theta_{0}\right)}^{\prime }\right)}^{-\frac{1}{2}}\frac{1}{\sqrt{T}}\overset{T}{\underset{t=2}{\sum }}M_{t}\left(\theta_{0}\right)\right]+o_{p}\left(1\right).$$

*It is easy to see that*

$$E\left(\frac{𝜕M_{t}\left(\theta_{0}\right)}{𝜕\theta }\right){\left[E\left(\frac{𝜕M_{t}\left(\theta_{0}\right)}{𝜕\theta^{\prime }}\right){\left(E\left(M_{t}\left(\theta_{0}\right)M_{t}{\left(\theta_{0}\right)}^{\prime }\right)\right)}^{-1}E\left(\frac{𝜕M_{t}\left(\theta_{0}\right)}{𝜕\theta }\right)\right]}^{-1}E\left(\frac{𝜕M_{t}\left(\theta_{0}\right)}{𝜕\theta^{\prime }}\right)$$

$$-\left(\frac{𝜕M_{t}\left(\theta_{0}\right)}{𝜕\theta_{\left(1\right)}^{\;}}\right){\left[E\left(\frac{𝜕M_{t}\left(\theta_{0}\right)}{𝜕\theta_{\left(1\right)}^{'}}\right){\left(E\left(M_{t}\left(\theta_{0}\right)M_{t}{\left(\theta_{0}\right)}^{'}\right)\right)}^{-1}E\left(\frac{𝜕M_{t}\left(\theta_{0}\right)}{𝜕\theta_{\left(1\right)}}\right)\right]}^{-1}E\left(\frac{𝜕M_{t}\left(\theta_{0}\right)}{𝜕\theta_{\left(1\right)}^{'}}\right)$$

*is a symmetric matrix; we will now show that this symmetric matrix is positive–semi definite:*

$$E\left(\frac{𝜕M_{t}\left(\theta_{0}\right)}{𝜕\theta }\right){\left[E\left(\frac{𝜕M_{t}\left(\theta_{0}\right)}{𝜕\theta^{\prime }}\right){\left(E\left(M_{t}\left(\theta_{0}\right)M_{t}{\left(\theta_{0}\right)}^{\prime }\right)\right)}^{-1}E\left(\frac{𝜕M_{t}\left(\theta_{0}\right)}{𝜕\theta }\right)\right]}^{-1}E\left(\frac{𝜕M_{t}\left(\theta_{0}\right)}{𝜕\theta^{\prime }}\right)$$

$$≳\left[\begin{array}{cc} E\left(\frac{𝜕M_{t}\left(\theta_{0}\right)}{𝜕\theta^{\;}}\right) & E\left(\frac{𝜕M_{t}\left(\theta_{0}\right)}{𝜕\theta_{\left(1\right)}^{\;}}\right) \end{array}\right]\left[\begin{array}{cc} E\left(\frac{𝜕M_{t}\left(\theta_{0}\right)}{𝜕\theta_{\left(1\right)}^{'}}\right){\left(E\left(M_{t}\left(\theta_{0}\right)M_{t}{\left(\theta_{0}\right)}^{'}\right)\right)}^{-1}E\left(\frac{𝜕M_{t}\left(\theta_{0}\right)}{𝜕\theta_{\left(1\right)}}\right) & 0\\ 0 & 0 \end{array}\right]\left[\begin{array}{c} E\left(\frac{𝜕M_{t}\left(\theta_{0}\right)}{𝜕\theta_{\left(1\right)}^{'}}\right)\\ E\left(\frac{𝜕M_{t}\left(\theta_{0}\right)}{𝜕\theta_{\left(2\right)}^{'}}\right) \end{array}\right]$$

$$=\left(E\frac{𝜕M_{t}\left(\theta_{0}\right)}{𝜕\theta_{\left(1\right)}}\right){\left[E\left(\frac{𝜕M_{t}\left(\theta_{0}\right)}{𝜕\theta_{\left(1\right)}^{\prime }}\right){\left(E\left(M_{t}\left(\theta_{0}\right)M_{t}{\left(\theta_{0}\right)}^{\prime }\right)\right)}^{-1}E\left(\frac{𝜕M_{t}\left(\theta_{0}\right)}{𝜕\theta_{\left(1\right)}}\right)\right]}^{-1}E\left(\frac{𝜕M_{t}\left(\theta_{0}\right)}{𝜕\theta_{\left(1\right)}^{\prime }}\right),$$

*here,* A≳B *implies that* A−B *is positive–semi definite. Therefore, by the result in Rao [32], we have:*

$${ℒ}_{E}\left(\theta_{0}^{\left(1\right)},{\tilde{\theta }}_{EL}^{\left(2\right)}\right)-{ℒ}_{E}\left({\hat{\theta }}_{EL}^{\left(1\right)},{\hat{\theta }}_{EL}^{\left(2\right)}\right)\overset{d}{\to }\chi^{2}\left(q\right).\;$$

### Appendix A.2. Complementary Numerical Simulations

**Table A1:** Parameter Estimation Simulation Results.

Sample Size	$\beta_{0}^{\left(CLS\right)}$	$\beta_{0}^{\left(CML\right)}$	$\beta_{1}^{\left(CLS\right)}$	$\beta_{1}^{\left(CML\right)}$	$\lambda^{\left(CLS\right)}$	$\lambda^{\left(CML\right)}$
$\mathrm{Parameter}:\;\beta_{0}=2$ $\beta_{1}=-0.8$, λ=3.5 $\phi_{t}|y_{t-1}$ is fixed.						
T = 300						
BIAS	0.4431	0.3667	−0.1879	−0.1624	−0.0282	−0.0306
RMSE	2.5257	2.7556	1.4189	1.1704	0.2811	0.2811
MAPE	0.4738	0.4791	0.3841	0.3682	0.0637	0.0637
T = 500						
BIAS	0.2098	0.2051	−0.0759	−0.0741	−0.0074	−0.0085
RMSE	0.8623	0.8619	0.4246	0.4371	0.2066	0.2062
MAPE	0.3049	0.3041	0.2148	0.2141	0.0475	0.0474
T = 800						
BIAS	0.1109	0.1071	−0.0346	−0.0329	−0.0119	−0.0126
RMSE	0.5673	0.5609	0.1646	0.1619	0.1783	0.1775
MAPE	0.2229	0.2208	0.1509	0.1496	0.0404	0.0403
T = 1200						
BIAS	0.0862	0.0848	−0.0232	−0.0224	−0.0093	−0.0101
RMSE	0.4491	0.4477	0.1201	0.1193	0.1375	0.1371
MAPE	0.1773	0.1771	0.1169	0.1163	0.0313	0.0311
T = 2000						
BIAS	0.0278	0.0269	−0.0119	−0.0115	0.0007	0.0003
RMSE	0.3369	0.3359	0.0889	0.0889	0.1076	0.1074
MAPE	0.1339	0.1333	0.0864	0.0864	0.0246	0.0244
$\mathrm{Parameter}:\;\beta_{0}=2$, $\beta_{1}=-0.8$, λ=3.5 $\phi_{t}|y_{t-1}$ follows a uniform distribution.						
T = 300						
BIAS	0.5624	0.3983	−0.2331	−0.1424	−0.0404	−0.0203
RMSE	2.0828	1.1916	1.2894	0.4719	0.2807	0.2534
MAPE	0.4877	0.4146	0.4355	0.3232	0.0641	0.0581
T = 500						
BIAS	0.1717	0.1399	−0.0712	−0.0593	−0.0028	0.0079
RMSE	0.8577	0.7919	0.3289	0.2552	0.2153	0.1982
MAPE	0.3028	0.2852	0.2125	0.2012	0.0496	0.0543
T = 800						
BIAS	0.1036	0.0809	−0.0317	−0.0285	−0.0124	−0.0039
RMSE	0.5735	0.5538	0.1547	0.1499	0.1725	0.1563
MAPE	0.2212	0.2158	0.1458	0.1427	0.0405	0.0036
T = 1200						
BIAS	0.0531	0.0367	−0.0152	−0.0131	−0.0114	−0.0067
RMSE	0.4479	0.4334	0.1217	0.1196	0.1445	0.1303
MAPE	0.1785	0.1723	0.1177	0.1167	0.0331	0.0301
T = 2000						
BIAS	0.0453	0.0385	−0.0143	−0.0129	−0.0048	−0.0029
RMSE	0.3493	0.3429	0.0912	0.0898	0.1091	0.0903
MAPE	0.1389	0.1354	0.0885	0.0871	0.0248	0.0231
$\mathrm{Parameter}:\;\beta_{0}=2$, $\beta_{1}=-0.8$, λ=3.5 $\phi_{t}|y_{t-1}$ follows an exponential distribution.						
T = 300						
BIAS	0.5805	0.4213	−0.2463	−0.1944	−0.0092	0.0232
RMSE	2.2029	2.0433	1.0969	1.0533	0.2702	0.2058
MAPE	0.5443	0.4843	0.4557	0.3986	0.0614	0.0466
T = 500						
BIAS	0.1923	0.0879	−0.0723	−0.0451	−0.0131	−0.0071
RMSE	1.0283	0.8006	0.2859	0.2364	0.2127	0.1601
MAPE	0.3299	0.2888	0.2236	0.1963	0.0483	0.0359
T = 800						
BIAS	0.1439	0.0929	−0.0464	−0.0336	−0.0061	0.0047
RMSE	0.6386	0.5709	0.1855	0.1605	0.1724	0.1293
MAPE	0.2456	0.2238	0.1653	0.1497	0.0389	0.0291
T = 1200						
BIAS	0.0699	0.0416	−0.0201	−0.0167	−0.0095	0.0025
RMSE	0.4731	0.4405	0.1242	0.1169	0.1404	0.1049
MAPE	0.1869	0.1744	0.1172	0.1123	0.0322	0.0239
T=2000						
BIAS	0.0519	0.0319	−0.0151	−0.0111	−0.0049	0.0007
RMSE	0.3669	0.3435	0.0976	0.9161	0.1106	0.0818
MAPE	0.1442	0.1369	0.0955	0.0908	0.0251	0.0185
$\mathrm{Parameter}:\;\beta_{0}=2$, $\beta_{1}=-0.8$, λ=3.5 $\phi_{t}|y_{t-1}$ follows a chi−square distribution.						
T = 300						
BIAS	0.9824	0.4063	−0.5663	−0.1282	−0.0098	0.0078
RMSE	3.3564	2.2437	1.6833	0.6341	0.3081	0.1569
MAPE	0.8569	0.5793	0.7361	0.3699	0.0696	0.0361
T = 500						
BIAS	0.4831	0.2249	−0.1805	−0.0621	−0.0202	−0.0068
RMSE	1.4549	0.9875	0.8114	0.2533	0.2293	0.1187
MAPE	0.4856	0.3749	0.3716	0.2354	0.0514	0.0269
T = 800						
BIAS	0.2344	0.0869	−0.092	−0.0305	−0.008	0.0036
RMSE	1.0181	0.7138	0.4998	0.1758	0.1916	0.0962
MAPE	0.3477	0.2792	0.2501	0.1712	0.0433	0.0221
T = 1200						
BIAS	0.1382	0.0428	−0.041	−0.015	−0.014	−0.0021
RMSE	0.6592	0.5481	0.1766	0.1351	0.1557	0.0782
MAPE	0.2531	0.2164	0.1649	0.1325	0.0353	0.0181
T = 2000						
BIAS	0.0751	0.0438	−0.0269	−0.0161	−0.0011	0.0019
RMSE	0.5081	0.4318	0.1322	0.1079	0.1211	0.0611
MAPE	0.2017	0.1713	0.1279	0.1061	0.0277	0.0141

**Table A2:** Simulation Results for Parameter Estimation under Model Misspecification. With likelihood function settled as $\phi_{t}|y_{t-1}$ it follows a chi-squared distribution.

Sample Size	$\beta_{0}^{\left(CLS\right)}$	$\beta_{0}^{\left(CML\right)}$	$\beta_{1}^{\left(CLS\right)}$	$\beta_{1}^{\left(CML\right)}$	$\lambda^{\left(CLS\right)}$	$\lambda^{\left(CML\right)}$
$\mathrm{Parameter}:\;\beta_{0}=1$, $\beta_{1}=-0.6$, λ=1.2 $\phi_{t}|y_{t-1}$ follows a uniform distribution.						
T = 300						
BIAS	0.0865	−1.8661	−0.0456	−1.5741	0.0046	0.4508
RMSE	0.8065	4.647	0.2301	5.3142	0.1454	0.4777
MAPE	0.6267	2.7518	0.2865	3.1747	0.0964	0.3757
T = 500						
BIAS	0.0312	−2.0474	−0.0228	−0.788	0.0043	0.4587
RMSE	0.5636	5.0468	0.1567	5.2847	0.1052	0.4753
MAPE	0.4493	2.5831	0.2046	1.9182	0.0703	0.3823
T = 800						
BIAS	0.0292	−2.1058	−0.0165	−0.3596	0.0038	0.4548
RMSE	0.4503	3.2688	0.1244	3.0257	0.0852	0.4657
MAPE	0.3587	2.3491	0.1651	1.2312	0.0563	0.3789
T = 1200						
BIAS	0.0249	−2.1077	−0.0127	−0.0739	0.0003	0.4558
RMSE	0.3513	2.6833	0.0971	1.7031	0.0689	0.4641
MAPE	0.2815	2.1461	0.1289	0.7674	0.0464	0.3799
T = 2000						
BIAS	0.0062	−2.0216	−0.0041	0.0766	0.0016	0.4546
RMSE	0.2735	2.2846	0.0749	1.0373	0.0529	0.4591
MAPE	0.2165	2.0256	0.0983	0.5483	0.0353	0.3788

**Table A3:** Empirical Likelihood Test for the λ Parameter. The significance level is set at 0.05.

Parameter: $\beta_{0}=1$, $\beta_{1}=-0.6$, λ=1.2 $\phi_{t}|y_{t-1}$ is fixed.					
T	300	500	800	1200	2000
${ℍ}_{0}:\lambda =1.5$	0.537	0.705	0.865	0.995	1
${ℍ}_{0}:\lambda =1.35$	0.235	0.263	0.375	0.542	0.757
${ℍ}_{0}:\lambda =1.2$ (true)	0.038	0.045	0.043	0.052	0.055
${ℍ}_{0}:\lambda =1.05$	0.176	0.33	0.415	0.593	0.823
${ℍ}_{0}:\lambda =0.9$	0.554	0.806	0.96	1	1
Parameter: $\beta_{0}=1$, $\beta_{1}=-0.6$, λ=1.2 $\phi_{t}|y_{t-1}$ follows a uniform distribution.					
T	300	500	800	1200	2000
${ℍ}_{0}:\lambda =1.5$	0.461	0.754	0.905	0.984	1
${ℍ}_{0}:\lambda =1.35$	0.212	0.304	0.417	0.54	0.786
${ℍ}_{0}:\lambda =1.2$ (true)	0.059	0.06	0.062	0.059	0.05
${ℍ}_{0}:\lambda =1.05$	0.167	0.321	0.407	0.588	0.845
${ℍ}_{0}:\lambda =0.9$	0.645	0.845	0.975	1	1
Parameter: $\beta_{0}=1$, $\beta_{1}=-0.6$, λ=1.2 $\phi_{t}|y_{t-1}$ follows an exponential distribution.					
T	300	500	800	1200	2000
${ℍ}_{0}:\lambda =1.5$	0.495	0.722	0.943	0.991	1
${ℍ}_{0}:\lambda =1.35$	0.171	0.31	0.505	0.593	0.844
${ℍ}_{0}:\lambda =1.2$ (true)	0.049	0.046	0.055	0.058	0.047
${ℍ}_{0}:\lambda =1.05$	0.235	0.286	0.442	0.605	0.884
${ℍ}_{0}:\lambda =0.9$	0.57	0.815	0.972	1	1
Parameter: $\beta_{0}=1$, $\beta_{1}=-0.6$, λ=1.2 $\phi_{t}|y_{t-1}$ follows a chi−square distribution.					
T	300	500	800	1200	2000
${ℍ}_{0}:\lambda =1.5$	0.478	0.648	0.852	0.951	1
${ℍ}_{0}:\lambda =1.35$	0.195	0.334	0.491	0.612	0.807
${ℍ}_{0}:\lambda =1.2$ (true)	0.086	0.088	0.079	0.054	0.051
${ℍ}_{0}:\lambda =1.05$	0.115	0.225	0.318	0.515	0.795
${ℍ}_{0}:\lambda =0.9$	0.417	0.635	0.859	0.946	1

**Table A4:** Empirical Likelihood Test for $\beta_{1}$ with a True Value of 0.

$\mathrm{Parameter}:\;\beta_{0}=1$, $\beta_{1}=0$, λ=1.2 $\phi_{t}|y_{t-1}$ follows an exponential distribution, significance level 0.05.					
T	300	500	800	1200	2000
${ℍ}_{0}:\beta_{1}=0$ (true)	0.437	0.446	0.416	0.51	0.427
${ℍ}_{0}:\beta_{1}=-0.1$	0.71	0.787	0.863	0.954	0.982
${ℍ}_{0}:\beta_{1}=-0.2$	0.813	0.933	0.989	0.997	1
${ℍ}_{0}:\beta_{1}=-0.3$	0.912	0.983	1	1	1
${ℍ}_{0}:\beta_{1}=-0.4$	0.945	0.982	1	1	1
$\mathrm{Parameter}:\;\beta_{0}=1$, $\beta_{1}=0$, λ=1.2 $\phi_{t}|y_{t-1}$ follows an exponential distribution, significance level 0.10.					
T	300	500	800	1200	2000
${ℍ}_{0}:\beta_{1}=0$ (true)	0.543	0.544	0.517	0.613	0.55
${ℍ}_{0}:\beta_{1}=-0.1$	0.797	0.846	0.93	0.982	0.993
${ℍ}_{0}:\beta_{1}=-0.2$	0.872	0.957	0.988	1	1
${ℍ}_{0}:\beta_{1}=-0.3$	0.945	1	1	1	1
${ℍ}_{0}:\beta_{1}=-0.4$	0.971	0.985	1	1	1
